# n-3 Docosapentaenoic acid-derived protectin D1 promotes resolution of neuroinflammation and arrests epileptogenesis

**DOI:** 10.1093/brain/awy247

**Published:** 2018-10-10

**Authors:** Federica Frigerio, Giulia Pasqualini, Ilaria Craparotta, Sergio Marchini, Erwin A van Vliet, Patrick Foerch, Catherine Vandenplas, Karin Leclercq, Eleonora Aronica, Luca Porcu, Kimberly Pistorius, Romain A Colas, Trond V Hansen, Mauro Perretti, Rafal M Kaminski, Jesmond Dalli, Annamaria Vezzani

**Affiliations:** 1 Department of Neuroscience, Mario Negri Institute for Pharmacological Research IRCSS, Milano, Italy; 2 Department of Oncology, Mario Negri Institute for Pharmacological Research IRCSS, Milano, Italy; 3 Department of (Neuro)Pathology, Academic Medical Center, University of Amsterdam, Amsterdam, The Netherlands; 4 UCB Biopharma SPRL, Braine l’Alleud, Belgium; 5 Stichting Epilepsie Instellingen Nederland (SEIN), Amsterdam, The Netherlands; 6 William Harvey Research Institute, Queen Mary University of London, London, UK; 7 School of Pharmacy, Department of Pharmaceutical Chemistry, University of Oslo, Oslo, Norway; 8 Centre for inflammation and Therapeutic Innovation, Queen Mary University of London, London, UK

**Keywords:** pro-resolving lipid mediators, epilepsy, comorbidities, ALX/FPR2, ChemR23/ERV1

## Abstract

Epilepsy therapy is based on drugs that treat the symptoms rather than the underlying mechanisms of the disease (epileptogenesis). There are no treatments for preventing seizures or improving disease prognosis, including neurological comorbidities. The search of pathogenic mechanisms of epileptogenesis highlighted that neuroinflammatory cytokines [i.e. interleukin-1β (IL-1β), tumour necrosis factor-α (Tnf-α)] are induced in human and experimental epilepsies, and contribute to seizure generation in animal models. A major role in controlling the inflammatory response is played by specialized pro-resolving lipid mediators acting on specific G-protein coupled receptors. Of note, the role that these pathways have in epileptogenic tissue remains largely unexplored. Using a murine model of epilepsy, we show that specialized pro-resolving mechanisms are activated by status epilepticus before the onset of spontaneous seizures, but with a marked delay as compared to the neuroinflammatory response. This was assessed by measuring the time course of mRNA levels of 5-lipoxygenase (*Alox5*) and 15-lipoxygenase (*Alox15*), the key biosynthetic enzymes of pro-resolving lipid mediators, versus *Il1b* and *Tnfa* transcripts and proteins. In the same hippocampal tissue, we found a similar delayed expression of two main pro-resolving receptors, the lipoxin A_4_ receptor/formyl peptide receptor 2 and the chemerin receptor. These receptors were also induced in the human hippocampus after status epilepticus and in patients with temporal lobe epilepsy. This evidence supports the hypothesis that the neuroinflammatory response is sustained by a failure to engage pro-resolving mechanisms during epileptogenesis. Lipidomic LC-MS/MS analysis showed that lipid mediator levels apt to resolve the neuroinflammatory response were also significantly altered in the hippocampus during epileptogenesis with a shift in the biosynthesis of several pro-resolving mediator families including the n-3 docosapentaenoic acid (DPA)-derived protectin D1. Of note, intracerebroventricular injection of this mediator during epileptogenesis in mice dose-dependently reduced the hippocampal expression of both *Il1b* and *Tnfa* mRNAs. This effect was associated with marked improvement in mouse weight recovery and rescue of cognitive deficit in the novel object recognition test. Notably, the frequency of spontaneous seizures was drastically reduced by 2-fold on average and the average seizure duration was shortened by 40% after treatment discontinuation. As a result, the total time spent in seizures was reduced by 3-fold in mice treated with n-3 DPA-derived protectin D1. Taken together, the present findings demonstrate that epilepsy is characterized by an inadequate engagement of resolution pathways. Boosting endogenous resolution responses significantly improved disease outcomes, providing novel treatment avenues.

## Teaser

Frigerio *et al*. report that pro-resolving mechanisms of neuroinflammation are dysregulated during epileptogenesis, thereby promoting a persistent neuroinflammatory response that contributes to seizure generation and cognitive deficits. Boosting endogenous resolution responses by administering a specific lipid mediator improves disease outcomes in a murine epilepsy model, suggesting a novel treatment avenue.

## Introduction

Epilepsy is a brain disease that affects around 65 million people worldwide (Devinski *et al.*, 2018). Epileptic seizures, the hallmarks of epilepsy, reduce the quality of life, increase the risk of death, and impose socio-economic burdens to affected people and society. Despite the availability of a significant number of anti-seizure drugs, ∼30% of newly diagnosed epilepsy patients are resistant to therapies (Kwan *et al.*, 2011; Loscher *et al.*, 2013). Moreover, there are no drugs able to prevent the onset or progression of epilepsy in patients exposed to epileptogenic injuries, such as status epilepticus, trauma, stroke or CNS infections. Thus, next generation therapies should target the mechanisms of epileptogenesis to either prevent the disease or improve its outcomes (Loscher *et al.*, 2013).

Neuroinflammation is a mechanism of epileptogenesis commonly induced in seizure-susceptible brain areas by differing brain injuries (Aronica *et al.*, 2017; Klein *et al.*, 2018). It persists in chronic epilepsy in both experimental animals and patients (Aronica and Crino, 2011; Butler *et al.*, 2016; Klein *et al.*, 2018). Neuroinflammation is characterized by increased cytokines, such as interleukin-1β (IL-1β), tumour necrosis factor-α (TNF-α), high mobility group box 1 (HMGB1), their cognate receptors and downstream effector molecules in glia, neurons and blood–brain barrier cellular components (Ravizza *et al.*, 2006, 2008, 2011; Boer *et al.*, 2008; Vezzani *et al.*, 2013). These inflammatory molecules significantly contribute to the generation and recurrence of spontaneous seizures (Vezzani *et al.*, 2011*a*, *b*; Terrone *et al.*, 2017) and to neurological comorbidities (Mazarati *et al.*, 2017) in animal models. There is evidence that the neuroinflammatory *milieu* persists in epilepsy because is not efficiently controlled by endogenous anti-inflammatory mechanisms (Ravizza *et al.*, 2006; Aronica *et al.*, 2007; Pernhorst *et al.*, 2013; Sun *et al.*, 2016), thereby contributing to dysfunction of brain cells and neuronal network hyperexcitability (Vezzani *et al.*, 2011*b*; Vezzani and Viviani, 2015). In support, pharmacological or genetic interventions for increasing the endogenous anti-inflammatory IL-1 receptor antagonist (IL-1Ra, encoded by *IL1RN*) drastically reduced the frequency of seizures in experimental models (De Simoni *et al.*, 2000; Vezzani *et al.*, 2000, 2002; Librizzi *et al.*, 2012; Bertani *et al.*, 2017; Walker *et al.*, 2017) and in humans (Jyonouchi, 2016; Kenney-Jung *et al.*, 2016; DeSena *et al.*, 2018), afforded neuroprotection (Noé *et al.*, 2013) and rescued neurological deficits (Mazarati *et al.*, 2017).

Specialized pro-resolving mediators (e.g. lipoxins, resolvins, protectins and maresins) are key molecules that mediate the active resolution of inflammation in peripheral tissues and in CNS (Serhan *et al.*, 2008). The biosynthesis of these mediators occurs through the stereoselective conversion of essential fatty acids, including eicosapentaenoic acid (EPA), docosahexaenoic acid (DHA) and n-3 docosapentaenoic acid (DPA), by lipoxygenase (LOX) and cyclooxygenase enzymes (Serhan *et al.*, 2017). The biological actions of these molecules are mediated by cognate G-protein coupled receptors, including the lipoxin A4 receptor/formyl peptide receptor 2 (ALX/FPR2) and the chemerin receptor (ChemR23/ERV1), to drive active resolution of inflammation. This is attained by reducing the expression of proinflammatory cytokines and chemokines, orchestrating the trafficking of innate and adaptive immune cells to the inflamed tissue, restoring the blood–brain barrier integrity (Cristante *et al.*, 2013) and increasing anti-inflammatory molecules (Serhan *et al.*, 2008).

Pro-resolving mediators exert beneficial actions in a range of preclinical models of peripheral organ dysfunction linked to inflammatory conditions (Romano *et al.*, 2015; Serhan *et al.*, 2017). In the CNS, neuroprotective actions of lipoxins and their synthetic analogues, or neuroprotectin D1 (NPD1) (Zhao *et al.*, 2011) have been reported in models of Alzheimer’s disease, ischaemia-reperfusion injury or neurotrauma where these molecules also alleviate the neurological symptoms (Marcheselli *et al.*, 2003; Wu *et al.*, 2011; Zhao *et al.*, 2011; Luo *et al.*, 2013; Hawkins *et al.*, 2014). In models of neuropathic pain lipoxins (Martini *et al.*, 2016), maresin 1 (Serhan *et al.*, 2012), resolvins (Xu *et al.*, 2010) and NPD1 (Xu *et al.*, 2013) showed analgesic properties (Romano *et al.*, 2015; Keppel Hesselink, 2017).

The ALX/FPR2 receptor and 15-LOX (*ALOX15*) are expressed in the hippocampus of patients with Alzheimer’s disease suggesting that resolution mechanisms might be active in human CNS diseases with a pathogenic neuroinflammatory component. Of note, reduced levels of lipoxin (LX)A_4_ in the same specimens suggest a potential alteration of this pathway (Wang *et al.*, 2015).

Recent studies demonstrate that DHA and the DHA-derived NPD1 reduced neuronal hyperexcitability evoked *in vivo* by electrical hippocampal kindling stimulation (Musto *et al.*, 2011) or injection of a chemoconvulsant in mice (Musto *et al.*, 2015). Moreover, n-3 polyunsaturated fatty acids (such as EPA, DHA) decrease evoked hippocampal neuronal excitability in slice preparations (Xiao and Li, 1999; Taha *et al.*, 2013*b*) and there is some evidence that they may have anticonvulsive effects in humans and animal models (Taha *et al.*, 2010, 2013*a*). However, the mechanisms elicited by these n-3 polyunsaturated fatty acids as well as the role of pro-resolving pathways in epilepsy remain to be elucidated.

In this study, we provide novel evidence of the temporal regulation of specialized pro-resolving mediator expression in the hippocampus during epileptogenesis, and compared them with the kinetics of the neuroinflammatory response in a mouse model of acquired chronic epilepsy (Iori *et al.*, 2017). We also show that lipid mediator levels apt to resolve the neuroinflammatory response are significantly altered during epileptogenesis, and describe the expression of pro-resolving receptors in mouse and human epileptogenic hippocampi versus control brain tissue. Finally, we determined the ability of a specific specialized pro-resolving mediator, n-3 DPA-derived protectin D1 (PD1_n-3 DPA_), in controlling the neuroinflammatory response during epileptogenesis. This intervention reduced neuroinflammation, improved the post-injury animal’s weight recovery, rescued their cognitive deficit and resulted in a significant decrease in both frequency and duration of epileptic seizures.

## Materials and methods

In accordance with the ARRIVE guidelines, procedures involving animals and their care were conducted in conformity with the institutional guidelines that are in compliance with national (D.L. n.26, G.U. March 4, 2014) and international guidelines and laws (EEC Council Directive 86/609, OJ L 358, 1, December 12, 1987, Guide for the Care and Use of Laboratory Animals, U.S. National Research Council, 1996), and were reviewed and approved by the intramural ethical committee.

### Experimental animals

Adult male C57/BL6N mice (30 g; Charles River) were used in all the experiments, except for one experiment where adult male NMRI mice (28–32 g; Charles River) were used (Supplementary material). C57/BL6N mice were housed at constant temperature (23 ± 1°C) and relative humidity (60 ± 5%) with a fixed 12-h light-dark cycle and free access to food and water. Mice were housed four per cage until surgery, and then kept one per cage in an enriched environment with nesting materials (Hutchinson *et al.*, 2005).

### Mouse model of epilepsy

Mice were implanted under 1.5% isoflurane anaesthesia with a 23-gauge cannula unilaterally positioned on top of the dura mater for the intra-amygdala injection of kainic acid [from bregma, mm: nose bar 0; anteroposterior (AP) −1.0, lateral (L) −2.8]. A bipolar Teﬂon-insulated stainless-steel depth electrode (60 µm outer diameter) was implanted in the dorsal hippocampus ipsilateral to the injected amygdala (from bregma, mm: nose bar 0; AP −1.8, L −1.5, −2.0 below dura mater). Additionally, a cortical surface electrode was implanted onto the somatosensory cortex in the contralateral hemisphere. One additional guide cannula was positioned on top of the dura mater (from bregma, mm: nose bar 0; AP 0, L −0.9) ipsilateral to the injected amygdala for intracerebroventricular injections (Franklin and Paxinos, 2008). Finally, two screw electrodes were positioned over the nasal sinus and the cerebellum, and used as ground and reference electrodes, respectively. Electrodes were connected to a multipin socket and secured to the skull by acrylic dental cement (Iori *et al.*, 2017).

One week after surgery, mice were connected to the EEG setup the day before beginning the experiment in order to record an EEG baseline for at least 24 h. Kainic acid (0.3 μg in 0.2 μl; Sigma-Aldrich, #K0250) was dissolved in 0.1 M phosphate-buffered saline (PBS, pH 7.4) and injected into the right basolateral amygdala in freely moving mice using a needle protruding of 4.1 mm below the implanted cannula in order to evoke status epilepticus (Iori *et al.*, 2017).

### Detection and quantification of status epilepticus and spontaneous seizures

Status epilepticus was defined by the appearance of continuous spike activity with a frequency >1 Hz intermixed with high amplitude and frequency discharges lasting for at least 5 s, with a frequency of >8 Hz. Spikes were defined as sharp waves with amplitude at least 2.5-fold higher than the standard deviation of baseline signal and duration <100 ms, or as a spike-and-wave with duration <200 ms (Pitkanen *et al.*, 2005). Status epilepticus developed after ∼10 min from kainic acid injection, as previously described (Iori *et al.*, 2017). After 40 min from status epilepticus onset, mice were injected with diazepam (10 mg/kg, intraperitoneally) to improve their survival, although EEG status epilepticus was not interrupted. After status epilepticus induction mice were recorded continuously (24/7) until the onset of spontaneous seizures was detected in each mouse (Fig. 6C) and for 16 days thereafter using the Twin EEG Recording System connected with a Comet AS-40 32/8 Amplifier (sampling rate 400 Hz, high-pass filter 0.3 Hz, low-pass filter 70 Hz, sensitivity 2000 mV/cm; Grass-Telefactor). Digitized EEG data were processed using the Twin record and review software. Status epilepticus duration and spiking activity were quantified using Clampfit 9.0 program (Axon Instruments). The end of status epilepticus was defined by the occurrence of interspike intervals longer than 1 s. Spontaneous motor seizures developed 5.4 ± 0.5 days after status epilepticus (Fig. 6C) as described previously (Jimenez-Mateos *et al.*, 2012; Iori *et al.*, 2017). We calculated the total number and total duration of seizures during a 16-day recording period (24/7) starting after the onset of the first spontaneous seizure in each mouse to estimate the frequency of seizures and their average duration. Although we did not record mice for a longer time, we showed previously in this model that all mice exposed to status epilepticus lasting for about 7 h develop spontaneous seizures that recur for more than 2 months, and seizure frequency may increase from 1.5 months onwards in a proportion of mice (Iori *et al.*, 2017).

### Lipid mediator profiling

Mice were deeply anaesthetized with intraperitoneal injections of ketamine (75 mg/kg) and medetomidine (0.5 mg/kg), then perfused via ascending aorta with 50 mM ice-cold PBS (pH 7.4) for 1 min to remove blood, and decapitated at 72 h post-status epilepticus. The hippocampus ipsilateral to the stimulated amygdala was rapidly dissected out at 4°C, immediately frozen in liquid nitrogen and stored at −80°C. To have enough tissue for the analysis, we pooled two hippocampi ipsilateral to the stimulated amygdala from two different mice (*n* = 16 mice; eight samples). Sham mice (*n* = 8) were injected with vehicle instead of kainic acid; for each mouse the hippocampi from both hemispheres were pooled together.

Tissues were placed in 1 ml of ice-cold methanol containing 500 pg each of d_5_-lipoxin A_4_ (LXA_4_), d_5_-resolvin D2 (RvD2), d_4_-prostaglandin E_2_ (PGE_2_), d_4_-leukotriene B_4_ (LTB_4_) and d_8_-5S-hydroexeicosatetraneoic acid (diHETE). The tissues were then gently homogenized using a glass dounce. These were then kept at −20°C for 45 min, to allow protein precipitation, then centrifuged at 1500 *g* for 10 min, 4°C. All samples for LC-MS/MS-based profiling were extracted using solid-phase extraction columns (Dalli *et al.*, 2013; Rathod *et al.*, 2017). Briefly, supernatants were subjected to solid phase extraction, methyl formate fraction collected, brought to dryness and suspended in phase (methanol/water, 1:4, vol/vol) for injection on a Shimadzu LC-20AD HPLC and a Shimadzu SIL-20AC autoinjector, paired with a QTRAP® 6500 plus (Sciex). An Agilent Poroshell 120 EC-C18 column (100 mm × 4.6 mm × 2.7 μm) was kept at 50°C and mediators eluted using a mobile phase consisting of methanol-water-acetic acid of 20:80:0.01 (vol/vol/vol) that was ramped to 50:50:0.01 (vol/vol/vol) over 0.5 min and then to 80:20:0.01 (vol/vol/vol) from 2 min to 11 min, maintained till 14.5 min and then rapidly ramped to 98:2:0.01 (vol/vol/vol) for the next 0.1 min. This was subsequently maintained at 98:2:0.01 (vol/vol/vol) for 5.4 min, and the flow rate was maintained at 0.5 ml/min. The QTRAP® 6500 plus was operated using a multiple reaction monitoring method (Dalli *et al.*, 2013; Rathod *et al.*, 2017). Each lipid mediator was identified using established criteria including matching retention time to synthetic materials and authentic material and at least six diagnostic ions (Dalli *et al.*, 2013; Rathod *et al.*, 2017). Calibration curves were obtained for each mediator using synthetic and authentic standard mixtures at 0.78, 1.56, 3.12, 6.25, 12.5, 25, 50, 100, and 200 pg that gave linear calibration curves with a r^2^-values of 0.98–0.99.

### Pharmacological treatment with PD1_n-3 DPA_-ME

PD1_n-3 DPA_-ME, the methylester (ME) pro-drug form of PD1_n-3 DPA_, which is rapidly converted by cellular esterases to the free acid form, was prepared as previously described (Aursnes *et al.*, 2014). This was aliquoted in ethanol and stored at −80°C in the dark until use. Immediately prior to administration in mice, ethanol was evaporated using a gentle stream of nitrogen. Then, the mediator was suspended in 50 mM PBS (pH 7.4), placed in a water bath sonicator for <10 s and vortexed for no more than 30 s.

Mice were exposed to status epilepticus, then they were assigned randomly to treatment or vehicle (50 mM PBS) groups. PD1_n-3 DPA_-ME (20 or 200 ng/μl in 50 mM PBS, pH 7.4) or vehicle was injected intracerebroventricularly (1 μl/site) twice daily for four consecutive days starting 1 h after status epilepticus onset. This schedule was designed to encompass the epileptogenesis phase preceding disease onset, then the treatment was stopped.

### Supplementary methods

The Supplementary material provides further details for: real-time quantitative polymerase chain reaction analysis (RT-qPCR); intraperitoneal pilocarpine injection in mice; novel object recognition test; immunohistochemistry and double-immunostaining; human subjects; ALXR/FPR2 and ChemR23/ERV1 immunohistochemistry in human tissue; and histological analysis and quantification of neuronal cell loss, neurogenesis and glia activation.

### Statistical analysis

In each animal experiment no *ad interim* analysis was done. Sample size was *a priori* determined based on previous experience with the animal model. All efforts were made to minimize the number of animals used and their suffering according to the principles of the three Rs (Replacement, Reduction and Refinement; https://www.nc3rs.org.uk/the-3rs). Endpoints (outcome measures) and statistical tests were prospectively selected. A simple random allocation using a website randomization program (www.randomization.com) was applied to assign a subject to a particular experimental group. Data acquisition and analysis were done blindly.

Statistical analysis was performed by GraphPad Prism 6 (GraphPad Software, USA) for Windows using absolute values. Data are presented as box-and-whisker plots depicting median, interquartile interval, minimum and maximum (*n* = number of individual samples). Mann Whitney U-test for two independent groups and Kruskal-Wallis test with Dunn’s *post hoc* correction for more than two independent groups were used for statistical analysis of data. The temporal distribution of spikes during status epilepticus was analysed by two-way ANOVA followed by Bonferroni’s multiple comparisons test. Based on normality test’s results, one-way ANOVA with Tukey’s *post hoc* test and *t*-test were used for behavioural data. One tailed *t*-test was used for analysis of the effect of treatment with PD1_n-3DPA_-ME on spontaneous seizures. Friedman’s two-way non-parametric ANOVA was used to detect the treatment effect on number of seizures and duration and their interaction with days. Differences were considered significant with a *P* < 0.05.

### Data availability

The data that support the findings of this study are available from the corresponding author, upon reasonable request.

## Results

### Distinct temporal regulation of pro-resolving mediators versus neuroinflammatory mediators during epileptogenesis

**Figure 1:**
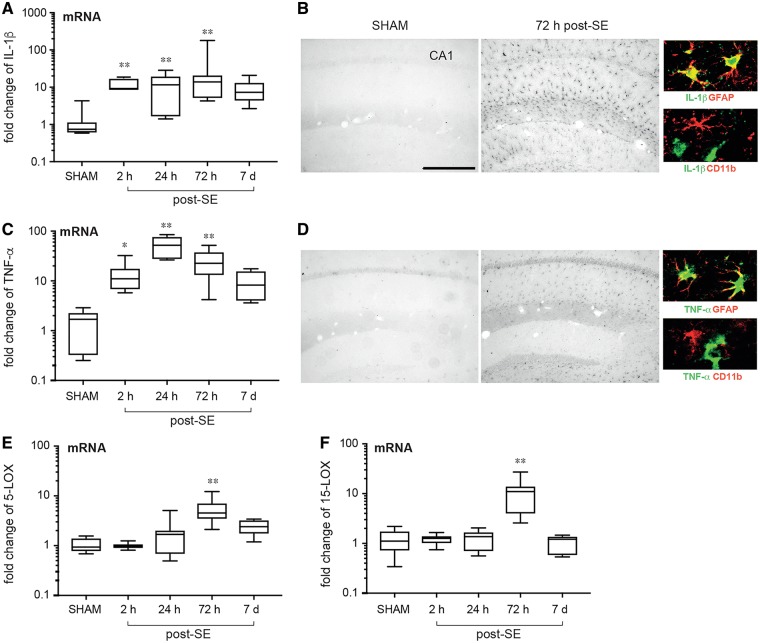
**Temporal regulation of pro-resolving pathways in mouse hippocampus during epileptogenesis.** Messenger RNA (**A** and **C**) and protein expression (**B** and** D**) of IL1B (IL-1β) and TNFA (TNF-α) and mRNA expression of *Alox5* (5-LOX, **B**) and *Alox15* (15-LOX, **F**) from 2 h until 7 days post-status epilepticus. Reference genes were *Mfsd5*, *Brap*, *Bcl2l13.* Data are presented on a log_10_ scale as box-and-whisker plots depicting median, interquartile interval, minimum and maximum (*n = *7–10). *P < 0.05, **P < 0.01 versus sham by Kruskal-Wallis test with Dunn’s *post hoc* correction. In the high magnification panels in **B** and **D**, we identified the cell types expressing IL-1β and TNF-α (green) by double-immunostaining using specific astrocytic (GFAP, red) and microglial (CD11b, red) cell markers. Co-localization signal is depicted in yellow. Scale bar = 50 μm.

As an index of neuroinflammation, we measured *Il1b* and *Tnfa* mRNA levels by RT-qPCR in the mouse hippocampus from 2 h until 7 days post-status epilepticus to encompass the epileptogenesis phase preceding the onset of spontaneous seizures. Figure 1A and C shows a significant increase in *Il1b* and *Tnfa* transcripts between 2 h and 72 h (*n* = 7–10) compared to sham mice (*n = *7), declining to basal values thereafter. The increase in cytokines was confirmed by immunohistochemistry (Fig. 1B and D) showing that both IL-1β and Tnf-α staining was induced in activated GFAP-positive astrocytes 72 h post-status epilepticus (*n = *8). No CD3-positive lymphocytes were observed in brain tissue (not shown). Cytokines were undetectable in control hippocampi (sham, *n = *8) or in CD11b-positive microglia 72 h after status epilepticus in mice (Fig. 1B and D, colour micrographs), which is in line with previous findings (De Simoni *et al.*, 2000; Maroso *et al.*, 2011).

To assess the potential impact of specialized pro-resolving mediators in these settings we determined the mRNA expression of *Alox5* (5-LOX) and *Alox15* (15-LOX), two key enzymes in the biosynthesis of pro-resolving mediators. In tissues from epileptogenic mice we found a significant upregulation of both enzymes at the 72 h interval (Fig. 1E and F; *n = *7–10 mice). To establish whether these results were also reproduced in a distinct model of epilepsy, we administered pilocarpine to mice and assessed the expression of both inflammatory and pro-resolving molecules in the hippocampus. We found a significant upregulation of both inflammatory and pro-resolving mediators tested at the 72 h interval (Supplementary Fig. 1). These results support the hypothesis that neuroinflammation in epileptogenesis may arise from a failure to engage pro-resolving mechanisms.

We also investigated the expression of two main pro-resolving receptors ALX/FPR2 and ChemR23/ERV1 in mouse (Supplementary Fig. 2A–D) and human (Supplementary Fig. 2E and F) epileptogenic tissues. In the mouse hippocampus the mRNA level of either receptor was increased above control value at 72 h (*n = *7) but not at 2 h and 7 days after status epilepticus (*n = *7–10). ALXR/FPR2 induction was measured at 24 h post-status epilepticus therefore anticipating ChemR23/ERV1 increase (Supplementary Fig. 2A and C). Both ALX/FPR2 and ChemR23/ERV1 immunostaining was increased in GFAP-positive astrocytes but not in CD11b-positive microglia 72 h post-status epilepticus (*n = *8) (Supplementary Fig. 2B and D, colour micrographs). ALX/FPR2 and IL-1β proteins were co-localized in astrocytes (micrograph in Supplementary Fig. 2B) suggesting that the resolution signalling process is operative within the cells that generate neuroinflammatory molecules. Similar to status epilepticus-exposed mice, ALX/FPR2 and ChemR23/ERV1 were predominantly induced in astrocytes in hippocampal specimens from patients who died 1–49 days after status epilepticus and in chronic epilepsy patients (*n = *7; Supplementary Fig. 2E and F) as compared to autoptic control tissue (*n = *6).

### Epileptogenesis alters mouse hippocampal specialized pro-resolving mediator profiles

**Figure 2:**
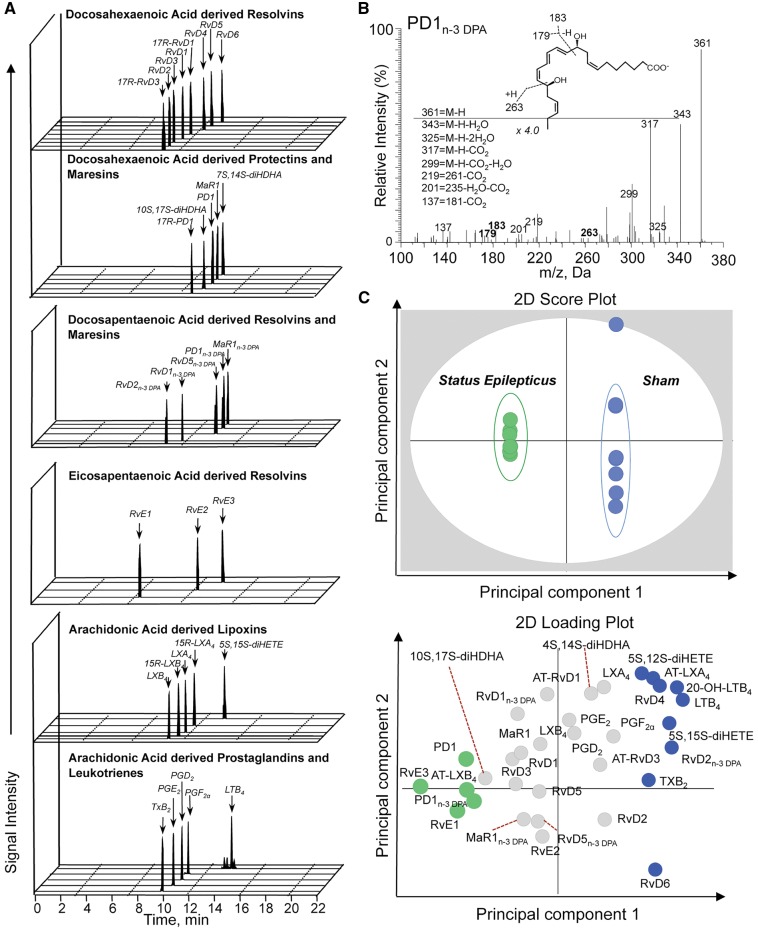
**Dysregulated lipid mediator profiles in hippocampus of mice during epileptogenesis.** Multiple reaction monitoring (**A**) for identified mediators in murine brains. (**B**) MS/MS spectrum employed for the identification of PD1_n-3 DPA_. (**C**) Octagonal-partial least square discriminant analysis of hippocampal lipid mediator profiles (*top*) 2D score plot; (*bottom*) 2D loading plot. Cumulative concentrations of bioactive mediator families identified in hippocampi from mice following status epilepticus and sham mice. Lipid mediators were isolated, identified and quantified using lipid mediator profiling in the hippocampus of mice 72 h post-status epilepticus (*n = *16) and in sham mice (*n = *8). See ‘Materials and methods’ section for details. (**C**) *P* < 0.05 sham versus status epilepticus using Mann Whitney U-test.

**Figure 3:**
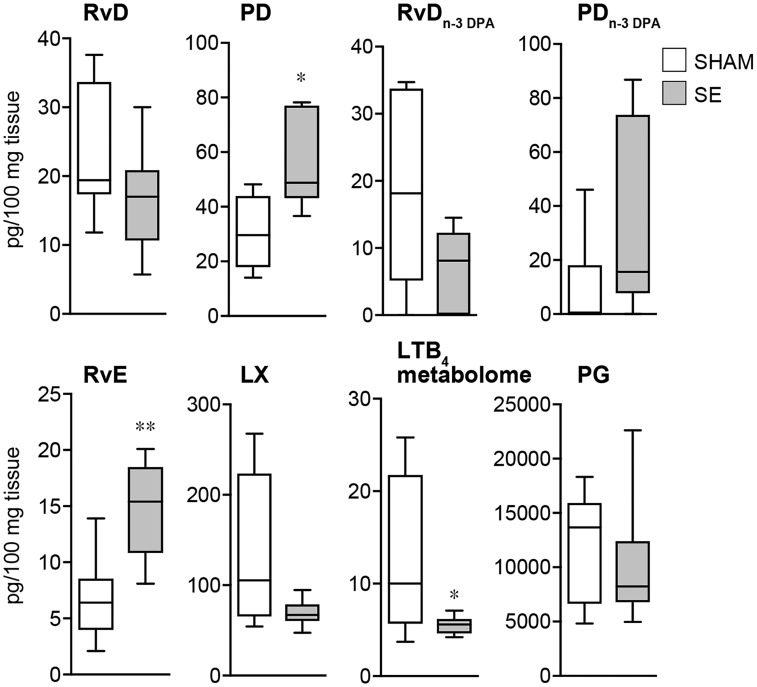
**Epileptogenesis alters specialized pro-resolving mediator profiles in the hippocampus. **The levels of DHA- and DPA-derived protectins and resolvins, EPA-derived resolvins and AA-derived lipoxins were identified and quantified using LC-MS/MS-based liquid mediator profiling (see ‘Materials and methods’ section for details) in the hippocampus of mice 72 h post-status epilepticus and in sham mice. Data are presented as box-and-whisker plots depicting median, interquartile interval, minimum and maximum (*n = *8 mice/group). RvD_n-3 DPA_ (*P* = 0.053); LX (*P* = 0.075); *P < 0.05; **P < 0.01 by Mann Whitney U-test.

Given that we found an alteration in resolution mechanisms and an upregulation in the expression of specialized pro-resolving mediator biosynthetic enzymes during murine epileptogenesis, we next assessed whether specialized pro-resolving mediator biosynthesis was also altered. Using LC-MS/MS-based lipid mediator profiling we identified mediators from all four major bioactive metabolomes that were identified in accordance to established criteria, including retention times and MS/MS spectra (Dalli *et al.*, 2018) (Fig. 2A and B). These included the DHA-derived resolvins and protectins and the n-3 DPA-derived protectins. Multivariate analysis of lipid mediator profiles obtained from hippocampi of sham mice and epileptogenic mice gave two distinct clusters indicating that lipid mediator production is dysregulated during epileptogenesis (Fig. 2C). Assessment of cumulative levels for the distinct specialized pro-resolving mediator families gave a downregulation of both DHA and n-3 DPA-derived resolvins and the arachidonic acid derived lipoxins. We also observed an upregulation of DHA and n-3 DPA derived protectins and eicosapentaenoic acid derived resolvins (Fig. 3). Assessment of individual mediator concentration gave statistically significant decreases in RvD2_n-3 DPA _and RvD5_n-3 DPA _as well as 5S,15S-diHETE, the lipoxin pathway marker, and an upregulation of PD1_n-3 DPA _in tissues from epileptogenic mice (Supplementary Table 2). These results demonstrate that epileptogenesis leads to dysregulation of specialized pro-resolving mediator production in the brain.

### PD1_n-3 DPA_ regulates neuroinflammation

**Figure 4:**
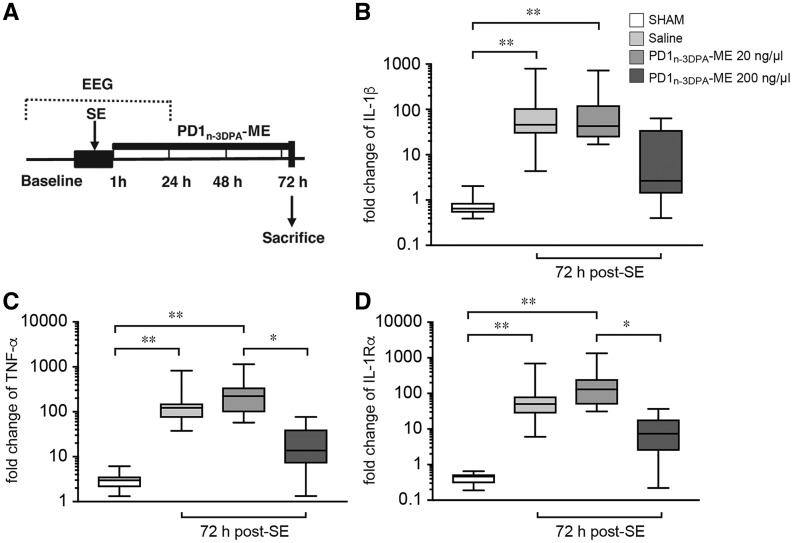
**PD1_n-3 DPA_ regulates components of the inflammatory response in the hippocampus during epileptogenesis.** (**A**) Experimental design applied to status epilepticus-exposed mice to study the effect of PD1_n-3 DPA_-ME on proinflammatory and anti-inflammatory mediators. One hour after status epilepticus onset, animals were randomized into a treatment arm injected intracerebroventricularly with either 20 ng/µl or 200 ng/µl PD1_n-3 DPA_-ME (*n = *6–9) and a saline-injected group (*n = *14). PD1_n-3 DPA_-ME or saline was injected for three consecutive days starting 1 h after status epilepticus onset. Mice were sacrificed 72 h post-status epilepticus (2 h after the last injection of PD1_n-3DPA_-ME or saline) for RT-qPCR analysis. Panels show mRNA levels of *Il1b* (IL-1β, **B**), *Tnfa* (TNF-α, **C**) *Il1rn* (IL-1Rα, **D**) in the hippocampus of mice treated with 20 ng/μl or 200 ng/μl dose of PD1_n-3 DPA_-ME (*n = *6–9) or saline (*n = *14) and in sham mice (*n = *12). Reference genes were *Mfsd5*, *Brap* and *Bcl2l13.* Data are presented on a log_10_ scale as box-and-whisker plots depicting median, interquartile interval, minimum and maximum (*n = *number of mice).*P < 0.05; ** *P* < 0.01 by Kruskal-Wallis test with Dunn’s *post hoc* correction.

Given that PD1_n-3 DPA_ was markedly upregulated during epileptogenesis, we next investigated whether this mediator was involved in controlling neuroinflammation. Thus, we administered this mediator as its methylester form (PD1_n-3 DPA_-ME), which is rapidly converted to the endogenous mediator in biological tissues. Administration of PD1_n-3 DPA_-ME (20 or 200 ng/µl) significantly reduced in a dose-dependent manner hippocampal *Il1b* and *Tnfa* mRNAs expression after status epilepticus when compared to mice administered vehicle only (Fig. 4). We also found that administration of this mediator reduced the expression of inflammation-dampening mediator IL-1Ra in brains from epileptogenic mice, likely because neuroinflammation was efficiently blunted by PD1_n-3 DPA_-ME. Of note, PD1_n-3 DPA_-ME administration did not modify status epilepticus onset, duration and severity (Supplementary Fig. 3). Together these results indicate that PD1_n-3 DPA _administration controls the onset and propagation of neuroinflammation during epileptogenesis.

### Effect of PD1_n-3 DPA _on early pathological consequences of status epilepticus

**Figure 5:**
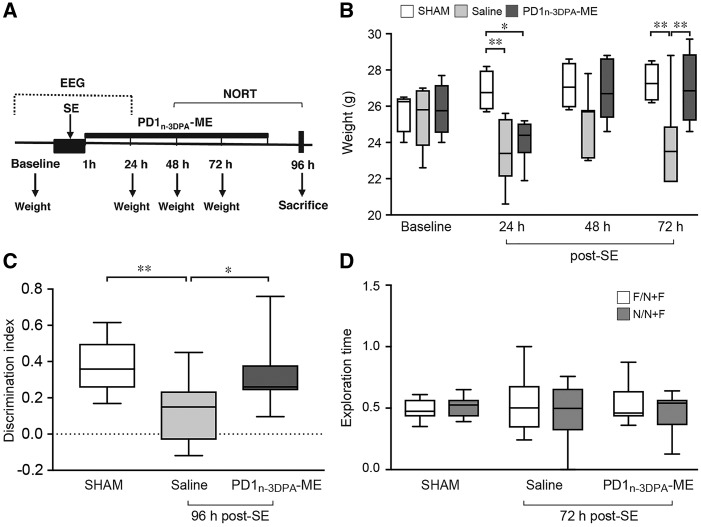
**PD1_n-3 DPA_ rescued weight loss and cognitive deficit during epileptogenesis.** (**A**) Experimental design applied to status epilepticus-exposed mice to study the effect of PD1_n-3 DPA_-ME on animal’s weight and recognition memory. One hour after status epilepticus onset, mice were randomized into PD1_n-3 DPA_-ME (200 ng/μl) or saline group. Mice were injected intracerebroventricular for four consecutive days starting 1 h after status epilepticus onset. (**B**) The weight of mice at baseline (the day before surgery) and at consecutive times after status epilepticus. Mice were weighed daily (between 8:00 and 9:00 am) after administering saline (*n = *7) or PD1_n-3 DPA_-ME (200 ng/μl; *n = *6). Sham mice (*n = *9). Data are presented as box-and-whisker plots depicting median, interquartile interval, minimum and maximum (*n = *number of mice). *P < 0.05; **P < 0.01 by Kruskal-Wallis test with Dunn’s *post hoc* correction. (**C** and **D**) The mice performance in the novel object recognition test (NORT) starting 48 h post-status epilepticus. (**C**) The discrimination index [the ratio between the difference in time spent with the novel and the familiar object (N−F) and the sum of total exploration time (N+F)]. The test was performed 96 h after status epilepticus in mice injected with saline (*n = *12) or PD1_n-3 DPA_-ME (*n = *11) and compared to sham mice (*n = *18). (**D**) The exploration time (the ratio between the time spent exploring the object and the total exploration time) during the familiarization phase when the two objects were identical. Data are presented as box-and-whisker plots depicting median, interquartile interval, minimum and maximum (*n = *number of mice). *P < 0.05; **P < 0.01 versus sham or saline by one-way ANOVA with Tukey’s *post hoc* test.

We next tested whether the protective mechanisms engaged by PD1_n-3 DPA _also translated to improvements in weight loss provoked by status epilepticus. Mice were administered PD1_n-3 DPA_-ME or vehicle intracerebroventricularly for 4 days starting 1 h after status epilepticus induction (Fig. 5A). Mice exposed to status epilepticus and injected with vehicle (*n = *7) displayed a persistent loss of weight after status epilepticus compared to sham mice (*n = *9; Fig. 5B). In contrast, in mice given PD1_n-3 DPA_-ME (*n = *6), weight returned to those in sham mice after 48 h and this effect was sustained through 72 h after status epilepticus (Fig. 5B). The baseline weight measured the day before surgery was similar in the three experimental groups (sham, 26.8 ± 0.3g; saline, 24.4 ± 0.4g; treatment, 25.9 ± 0.6g).

To determine whether there PD1_n-3 DPA_ also improved cognitive function, we performed the novel object recognition test (NORT) as a measure of non-spatial recognition memory. During the recognition phase of the NORT, control mice (*n = *18) spent significantly more time exploring the novel object as compared to the familiar one (∼70% and 30% of total exploration time, respectively), thus yielding a discrimination index of 0.37 ± 0.03 (Fig. 5C). By contrast, mice injected with saline and exposed to status epilepticus (*n = *12) spent equal time exploring previously presented and novel objects; hence the discrimination index was 0.13 ± 0.05 (Fig. 5C). This deficit was significantly rescued in mice treated with PD1_n-3 DPA_-ME (*n = *11) since they spent more time exploring the novel object thus yielding a discrimination index similar to control mice (Fig. 5C). The deficit in the NORT was not a result of changes in spontaneous motor activity since the distance covered and the velocity of mice in the open field were not affected by status epilepticus or the treatment (sham, 3061 ± 192 cm; 5.1 ± 0.3 cm/s; saline, 3364 ± 574 cm; 5.3 ± 0.6 cm/s; PD1_n-3 DPA_-ME, 3890 ± 613 cm; 6.9 ± 1.2 cm/s). As depicted in Fig. 5D, the three groups of mice spent equal time (∼50%) exploring the two identical objects during the familiarization phase, therefore excluding that the changes in discrimination index were due to different objects’ exploration times.

At the end of the behavioural test, the mice were killed for histopathological brain analysis. We did not detect significant differences in any of the cellular hallmarks measured in status epilepticus-exposed mice treated with PD1_n-3 DPA_-ME versus the corresponding vehicle-injected mice (Supplementary Fig. 4). In particular, there was an attenuation of the increased number of ectopic doublecortin-positive cell in the hilus (Supplementary Fig. 4A) as well as of astrogliosis and microgliosis (Supplementary Fig. 4B) in status epilepticus-exposed mice treated with by PD1_n-3 DPA_-ME versus vehicle, although this effect did not reach statistical significance. We measured a significant neuronal cell loss in the hippocampus (CA1, CA3) and the kainic acid-injected amygdala as assessed by counting Nissl-stained and Fluoro-Jade-positive neurons in status epilepticus-exposed mice treated with vehicle (Supplementary Fig. 4C and D). No significant neuroprotection was measured in PD1_n-3 DPA_-ME versus vehicle injected status epilepticus-exposed mice (Supplementary Fig. 4C and D).

### PD1_n-3 DPA _reduced ensuing epileptic seizures

**Figure 6:**
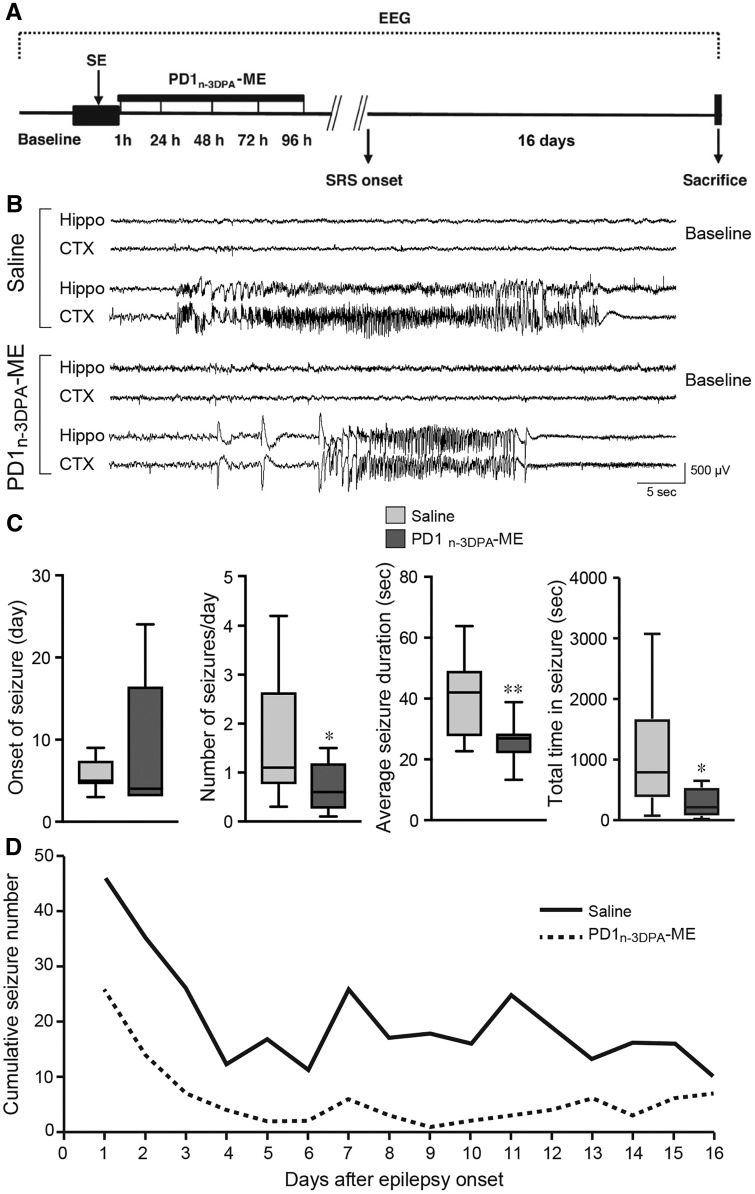
**PD1_n-3 DPA_ reduced spontaneous seizures.** (**A**) Experimental design in status epilepticus-exposed mice to study the effect of PD1_n-3 DPA_-ME on spontaneous seizures. One hour after status epilepticus induction, animals were randomized into treatment (PD1_n-3DPA_ME, *n = *9) and saline groups (*n = *12). PD1_n-3 DPA_-ME (200 ng/μl) or saline was injected intracerebroventricularly for four consecutive days starting 1 h after status epilepticus onset. EEG recording was done continuously from status epilepticus induction until the onset of the first two spontaneous seizures (SRS) and for 16 days thereafter. At the end of EEG recording mice were sacrificed for brain histological analysis. Representative EEG tracings depict baseline activity and spontaneous seizures recorded in the right hippocampus (Hippo) and in the left parietal cortex (CTX) of mice treated with saline or PD1_n-3 DPA_-ME (**B**). Note the shorter duration of a typical EEG seizure in a drug-treated mouse versus saline-injected mouse. (**C**) The onset of spontaneous seizures, the number of spontaneous seizures in each day of EEG recording, the average seizure duration and the cumulative time spent in seizures in saline- (*n = *12) versus drug-treated mice (*n = *9). Data are presented as box-and-whisker plots depicting median, interquartile interval, minimum and maximum (*n = *number of mice). (**D**) The cumulative number of seizures per day in each experimental group during 16 days after epilepsy onset. *P < 0.05; **P < 0.01 versus saline injected mice by one-tailed *t*-test. Friedman’s two-way non-parametric ANOVA was used to detect the treatment effect on number of seizures and duration and their interaction with days. Treatment effect on number of seizures, *P* < 0.01 and interaction with days, *P* = 0.51; treatment effect on duration of seizures, *P* < 0.01 and interaction with days, *P* = 0.82.

To test whether PD1_n-3 DPA _also regulates spontaneous seizures in mice, we administered the PD1_n-3 DPA_-ME (*n = *9) or saline (*n = *12) for 4 days starting 1 h after status epilepticus induction and cortical and hippocampal EEG activities were measured continuously for 21 days to detect the onset and frequency of spontaneous seizures (Fig. 6A and B). Although the onset of spontaneous seizures was not significantly modified by PD1_n-3 DPA_-ME (Fig. 6C), the mediator significantly reduced the cumulative number of daily seizures after disease onset which is reflected by an average 50% reduction of daily seizure frequency (Fig. 6C and D). The mean seizure duration was also significantly reduced in treated mice versus vehicle mice (Fig. 6B and C). As a result, the total time spent in seizures was reduced by 3-fold in mice treated with PD1_n-3 DPA_-ME (Fig. 6C). The reduction in the average seizure duration persisted at Day 16 from epilepsy onset in PD1_n-3 DPA_-ME-treated mice versus vehicle mice (vehicle, 41.4 ± 3.8 s; PD1_n-3 DPA_-ME, 30.1 ± 2.2, *P* < 0.05) despite the cumulative number of seizures at this day was similar in the two experimental groups (Fig. 6D).

The extent of neuronal cell loss in the hippocampus and amygdala in the epileptic mice treated with PD1_n-3 DPA_-ME during epileptogenesis was similar to vehicle-injected mice (Supplementary Fig. 5), in accordance with the evidence at 96 h post-status epilepticus (Supplementary Fig. 4C). We did not detect Fluoro-Jade-positive neurons in the epileptic mice at the end of EEG recordings indicating that neurodegeneration was complete.

Supplementary results are included in the Supplementary material.

## Discussion

Neuroinflammation is ignited with a rapid onset by various epileptogenic insults in both humans and animal models and persists in brain areas where seizures originate and spread (Gershen *et al.*, 2015; Butler *et al.*, 2016; Koepp *et al.*, 2017; Terrone *et al.*, 2017; van Vliet *et al.*, 2018). We reasoned that the dynamics of this response might point to inefficient mechanisms of resolution in the diseased brain tissue. Hence, lack of resolution may be a key factor for the pathological consequences of neuroinflammation in epilepsy (Vezzani *et al.*, 2015).

We tested the novel hypothesis that the active pro-resolving brain response mediated by fatty acid derivatives is inefficient in epilepsy, thereby contributing to persistent neuroinflammation and the consequent seizure generation, neuropathology and neurological deficits. We focused our investigations in the hippocampus, a key epileptogenic area, in a well-established murine model of epileptogenesis where combinations of anti-inflammatory drugs were proven to block disease progression (Iori *et al.*, 2017).

Similar to other epileptogenesis models (De Simoni *et al.*, 2000; Dhote *et al.*, 2007; Maroso *et al.*, 2011; Jiang *et al.*, 2013; Walker *et al.*, 2017), we found an early and lasting post-injury induction of the ictogenic cytokines IL-1β and TNF-α (Vezzani *et al.*, 2011*b*; Balosso *et al.*, 2013). Notably, we also determined the lack of activation of LOX enzymes and specialized pro-resolving mediator for hours to days post-insult, indicating a delayed and transient activation of the pro-resolving response as compared to neuroinflammation. Lipid mediator profiling demonstrated differential regulation in pro-resolving mediator concentrations during epileptogenesis, that is characteristic of a failed resolution response (Serhan, 2007, 2017), thus indicating that neuroinflammation contributing to epilepsy may arise from an inability of the neuronal tissue to engage these host protective pathways leading to tissue damage and neuronal impairment.

Notably, PD1_n-3 DPA_ was the most abundant lipid mediator during epileptogenesis suggesting it might be a key player in the tissue attempt to engage resolution pathways. Accordingly, early post-injury administration of PD1_n-3 DPA_-ME significantly reduced neuroinflammation in our murine model, in line with previous findings that PD1_n-3 DPA _and its precursor n-3 DPA (Ferdinandusse *et al.*, 2001) exert powerful anti-inflammatory effects (Dalli *et al.*, 2013). This effect was associated with a drastic reduction in the number and duration of spontaneous seizures after the onset of the disease. Since PD1_n-3 DPA_-ME therapeutic effect occurred after treatment termination, we conclude that the drug mediates a genuine anti-epileptogenic action rather than providing a mere symptomatic control of seizures. Importantly, PD1_n-3 DPA_-ME improved animal’s weight recovery after status epilepticus and rescued cognitive deficit, a major neurological comorbidity in epilepsy (Mazarati *et al.*, 2017). Since neuroinflammation contributes to both seizures and cognitive dysfunctions (Aronica *et al.*, 2017; Mazarati *et al.*, 2017), the anti-inflammatory properties of PD1_n-3 DPA_-ME are likely to chiefly mediate its therapeutic effects. An unavoidable limitation of the murine model is the short latency for the development of spontaneous seizures; therefore, the treatment necessarily overlaps with the status epilepticus. We have carefully quantified status epilepticus onset, severity and duration and these parameters were not changed by the early treatment with PD1_n-3 DPA_-ME. Moreover, PD1_n-3 DPA_-ME did not reduce neuronal cell loss in the hippocampus which is a direct consequence of status epilepticus. Nevertheless, one cannot exclude there might be a treatment effect on some acute modifications induced by status epilepticus, which play a role in the therapeutic outcomes.

n-3 DPA is known to reduce the activation of microglia, sphingomyelinase, caspase-3 and oxidative stress in aged rats, and as a consequence to attenuate age-related deficit in spatial learning and long-term potentiation (Kelly *et al.*, 2011). Similarly, the DHA-derived NPD1 inhibited these molecular processes in a model of Alzheimer’s disease (Zhao *et al.*, 2011) and also reduced hippocampal excitability and seizures in rodents (Musto *et al.*, 2011, 2015). Since these molecular events are strictly associated with neuroinflammation and play a role in seizure generation (Balosso *et al.*, 2008; Aronica *et al.*, 2017; Pauletti *et al.*, 2017), they may be implicated in the antiepileptogenic effects of PD1_n-3 DPA_-ME.

Histopathological brain evaluation in PD1_n-3 DPA_-ME-treated mice indicate a mild reduction in dentate hilus ectopic neurogenesis and in glial cells activation which are both implicated in epileptogenesis (Scharfman and McCloskey, 2009), but no neuroprotection was observed in forebrain. Differently, n-3 DPA exerted neuroprotective effects in the hippocampus of aged rats after 56 days of diet supplementation (Kelly *et al.*, 2011), suggesting that a more prolonged post-injury treatment with PD1_n-3 DPA_-ME is required to attain neuroprotective effects.

Overall our data support the concept that resolution of neuroinflammation and therapeutic effects on seizures and cognition can be attained by enhancing brain endogenous pro-resolving mediators. A potential clinical approach is to increment the dietary intake of omega-3-polyunsaturated fatty acids, which are known to be beneficial for health (Cash *et al.*, 2014). Animal studies suggest that n-3 polyunsaturated fatty acids raise the seizure threshold but the clinical studies have provided so far limited effects. This might be because of insufficient doses or too short periods of administration (Taha *et al.*, 2010). Long-term supplementation may be required to produce high enough levels of the active lipids in the brain. An advantage of this approach is the safety profile of diet supplementation. Since we observed induction of pro-resolving receptor expression in the hippocampus of patients who had status epilepticus or with drug-resistant seizures, treatments that enhance the availability of endogenous receptor ligands might be warranted. An alternative approach is to develop stable analogues of specialized pro-resolving mediator, including PD1_n-3DPA_, to overcome the limitation that they are metabolically unstable. A notable example is the proven efficacy in a phase II clinical trial of a stable analogue of resolvin E1 to treat dry eye symptoms. In this frame, our new evidence of the anti-epileptogenic effect of PD1_n-3 DPA_-ME highlights novel opportunity for drug discovery. However, the brain penetration of these potential new drugs should be improved for their therapeutic application in epilepsy, unless intrathecal application is conceived. These drugs might in principle be used as adjunctive therapy in patients exposed to epileptogenic injuries or with first presentation of seizures to decrease the burden of the disease and ameliorate its clinical course.

## Supplementary Material

Supplementary Data

## Abbreviations

DHA: docosahexaenoic acid
DPA: n-3 docosapentaenoic acid
LOX: lipoxygenase
PD1_n-3 DPA_: n-3 DPA-derived protectin D1
