# Supplementary material for: n-3 Docosapentaenoic acid-derived protectin D1 promotes resolution of neuroinflammation and arrests epileptogenesis
Source: Brain. 2018 Oct 10;141(11):3130–43. doi: 10.1093/brain/awy247 (PMC6202571; doi:10.1093/brain/awy247)
Supplement: Supplementary Data [file awy247_suppl_data.pdf]

Supplementary information

n-3 docosapentaenoic acid-derived protectin D1 promotes resolution of neuroinflammation and arrests epileptogenesis

Federica Frigerio[1], Giulia Pasqualini[1], Ilaria Craparotta[2], Sergio Marchini[2], Erwin A. van Vliet[3], Patrick Foerch[4], Catherine Vandenplas[4], Karin Leclercq[4], Eleonora Aronica[3,5], Luca Porcu[2], Kim Pistorius[6], Romain A. Colas[6], Trond V. Hansen[7], Mauro Perretti[6,8], Rafal M. Kaminski[4*], Jesmond Dalli[6,8*] and Annamaria Vezzani[1]*

[1]Department of Neuroscience and [2]Department of Oncology, IRCSS-Mario Negri Institute for Pharmacological Research, Milano, Italy; [3]Department of (Neuro)Pathology, Academic Medical Center, University of Amsterdam, Amsterdam, The Netherlands; [4]UCB Biopharma SPRL, Braine l'Alleud, Belgium; [5] Stichting Epilepsie Instellingen Nederland (SEIN); [6]William Harvey Research Institute, Queen Mary University of London, London, UK.; [7] School of Pharmacy, Department of Pharmaceutical Chemistry, University of Oslo, PO Box 1068 Blindern, N-0316 Oslo, Norway; [8]Centre for inflammation and Therapeutic Innovation, Queen Mary University of London, London, UK

Running title: *Proresolving lipids in epileptogenesis*

Supplementary methods

Real-time quantitative polymerase chain reaction analysis (RT-qPCR)

In a first experiment, intra-amygdala kainic acid-injected mice were sacrified 2 h, 24 h, 72 h, 7 days (n=7-10 mice/time point) after SE induction. In a second experiment, intra-amygdala kainic acid-injected mice treated with $PD1_{n\text{-}3DPA}$ME (20 or 200 ng/µl) were sacrified 72 h post-SE (n=6-9). Saline-injected mice were exposed to SE and served as control group for drug-treated mice (n=14). Sham mice were similarly implanted but were injected with vehicle and not exposed to SE (n=7-12). One additional group of mice was sacrified 72 h after SE induced by pilocarpine (n=10) and compared to respective sham mice (n=7).

Mice were deeply anesthetized with i.p. injections of ketamine (75 mg/kg) and medetomidine (0.5 mg/kg), then perfused via ascending aorta with 50 mM ice-cold PBS (pH 7.4) for 1 min to remove blood, and decapitated. The hippocampus ipsilateral to the stimulated amygdala was rapidly dissected out at 4°C in a RNAse free environment, immediately frozen in liquid nitrogen and stored at –80°C until assay. For RNA isolation, frozen hippocampi were homogenized in Qiazol Lysis Reagent (Qiagen Benelux, Venlo, The Netherlands, #79306). The total RNA including the miRNA fraction was isolated using the miRNeasy Mini kit (Qiagen Benelux, Venlo, the Netherlands #217004) according to manufacturer's instructions. The concentration and purity of RNA were determined at 260/280 nm using a high-speed microfluidic UV/VIS spectrophotometer QIAxpert (Qiagen, Milano, Italy) and the integrity and quality of RNA were evaluated by 4200 Tapestation (Agilent Technologies, Santa Clara, CA, USA).

cDNA was synthesized from 1000 ng RNA using the high capacity cDNA reverse transcription kit (Applied Biosystems, California, USA) following the manufacturer's protocol (Applied Biosystems, California, USA, #4368814). Each RT-qPCR analysis was run in triplicate for each experiment (the mean value of each sample was used for statistical analysis) in 384-well reaction plates in an automatic liquid handling station (epMotion 5075LH, Eppendorf, Hamburg, Germany) using an Applied Biosystems 7900HT System (Applied Biosystems, California, USA). cDNA was analyzed using Applied Biosystems TaqMan gene expression assays (*see table below*) according to protocol instructions.

Gene	Code	Amplicon Length
Mfsd5	Mm00547674	64
Brap	Mm00518493	82
Bcl2l13	Mm00463355	62
Il1b	Mm00434228	90
Il1rn	Mm00446186	80
Tnf	Mm00443260	61
Anxa1	Mm00440225	61
Il10	Mm01288386	136
Alxr	Mm00484464_s1	63
ChemR23	Mm02619757_s1	127

CT values were obtained using manual threshold and baseline, analyzed using the $2^{-\Delta\Delta Ct}$ method and normalized using geometric mean of 3 independent house-keeping genes (*Mfsd5*, *Brap*, *Bcl2l13*); Results are shown in Figures 1, 4 and Suppl. Fig. 1 and Suppl. Fig. 2.

Pilocarpine injection in mice

Adult male NMRI mice were used. Pilocarpine mice were generated as described previously (Mazzuferi *et al.*, 2012). Pilocarpine (300 mg/kg, Sigma-Aldrich) was intraperitoneally (i.p.) injected 30 min after i.p. administration of 1 mg/kg of N-methylscopolamine bromide (Sigma-Aldrich). Behavioral endpoints, such as the time to onset and the duration of SE as well as the survival rate were assessed. SE typically appeared within the first hour after pilocarpine injection (59.3 ± 2.6 min) and was characterized by continuous stage 3 to 5 motor seizures (Racine, 1972) without regaining consciousness (unresponsiveness to any environmental stimuli) together with loss of postural control. This definition is consistent with that being commonly used in the rat pilocarpine model (Turski *et al.*, 1983; Cavalheiro *et al.*, 1987). SE motor episodes were stopped after 2 h with an acute i.p. injection of diazepam (10 mg/kg). Sham mice were subjected to a similar schedule of injections, but with saline instead of pilocarpine. All mice injected with pilocarpine and their controls were killed 72 h post-SE for hippocampi dissection and RTqPCR analysis.

Novel object recognition test

We employed this behavioral test (NORT) to assess the ability of rodents to recognize a set of novel objects in an otherwise familiar environment since this behavior is taken as a measure of recognition memory (Balducci *et al.*, 2010). This task is predominantly associated with limbic cortex function although the 24 h inter-trial interval chosen from familiarization to test phase also

involves hippocampal activity (Balducci *et al.*, 2010; Mazarati *et al.*, 2011). Recognition memory was tested in mice exposed to SE and treated i.c.v. for 4 days with 200 ng/µl PD1$_{n\text{-}3DPA}$ME (n=11) or saline (n=12). Control mice (sham) were similarly implanted, injected with vehicle but not exposed to SE (n=18). Each mouse was weighted between 8:00 and 9:00 a.m. the day before surgery (baseline value), then 24 h, 48 h and 72 h post-SE. The test was performed for 3 consecutive days starting at 48 h after SE induction. At the end of the last session mice were sacrified (i.e. 96 h after SE) for histopathological analysis.

The test was performed in the open-square gray arena (40×40 cm) surrounded by 30-cm high wall, with the floor divided into 25 equal squares by black lines. Mouse behavior was remotely monitored via video camera. All experiments were started between 9:00 and 10:00 am. Twenty-four hours *prior* to the test, mice were allowed to habituate in the arena for 5 min (*habituation phase*). The test began on the next day with the *familiarization phase*, when mice were placed into the open field for 10 min in the presence of two identical objects positioned in internal nonadjacent squares. The following objects were randomly used: black plastic cylinders (4×5 cm); transparent scintillation vials with white cups (3×6 cm); metal cubes (3×5 cm). Cumulative exploration time of both objects and of each object separately was recorded. Exploration was defined as sniffing, touching, and stretching the head toward the object at a distance of not more than 2 cm. Twenty four hours after familiarization, the *recognition phase* of the test was performed: mice were placed for 10 min in the open field which contained one object presented during the familiarization phase (familiar object, F) and a novel unfamiliar object (N). The time spent exploring N vs F, as well as cumulative exploration time (i.e. novel+familiar, N+F) was recorded. As the recognition phase was performed 24 h after the familiarization phase, the procedure can be regarded as a test of long-term memory. Novel object recognition was quantified using the discrimination index (N-F/N+F): the difference between the time spent exploring the novel and the familiar objects (N-F) divided by the sum of total exploration time (N+F) (Okun *et al.*, 2010). At the end of NORT, all mice were sacrificed for histopathological analysis (*see below*).

Immunohistochemistry and double-immunostaining

Mice were sacrificed 72 h after SE onset for histological analysis (n=8) (Fig. 1). Control mice (sham) were implanted with electrodes and guide cannulae and injected with vehicles but were not exposed to SE (n=8). Mice were deeply anaesthetized by injecting ketamine (75 mg/kg, i.p.) and medetomidine (0.5 mg/kg, i.p.), then perfused via ascending aorta with 50 mM ice-cold PBS, pH 7.4 followed by chilled 4% paraformaldehyde (Merck, Darmstadt, Germany, #104005) in PBS. The brains were post-fixed for 90 min at 4° C, then transferred to 20% sucrose in PBS for 24 h at 4° C. Then, the brains were immersed in -45°C isopentane for 3 min and stored at -80°C until assayed.

Serial coronal sections were cut on a cryostat throughout the septo-temporal extension of the hippocampus (Franklin and Paxinos, 2008) (-0.94 to -3.64 mm *from bregma*). Four series of 16 sections each brain were prepared and in 3 series the slices were stained as follows: the 1st slice for IL-1β, the 2nd for TNF-α, the 4th for ALXR and the 5th for ChemR23. Three slices per mice were used for each marker. The same anatomical structures were retained within each series of sections to be compared.

IL-1β (1:200, Santa Cruz Bio, CA, USA, #sc-1252) and **TNF-α** (1:1000, Peprotech, NJ, USA, #500-P64G) immunohistochemistry was performed as previously described (De Simoni *et al.*, 2000; Maroso *et al.*, 2011).

For detection of **ALXR/FPR2 and ChemR23/ERV1** (Suppl. Fig. 2B,D), free-floating sections were rinsed for 15 min in FBS 3%, BSA 10% in 100 mM PBS at 4° C, followed by overnight incubation with the primary antibody anti-ALXR/FPR2 (1:3000, Novus Biologicals, CO, USA, #NLS1878) or anti-ChemR23/ERV1 (1:200, Santa Cruz, CA, USA, #SC-32652) at 4°C in 3% FBS and 10% BSA diluted in PBS.

Immunoreactivity was tested by the avidin-biotin-peroxidase technique (Vector Labs, Burlingame, CA, USA, #PK6100) using 3',3'-diaminobenzidine (DAB, Sigma-Aldrich, Munich, Germany, #D8001) as chromogen, and the signal was amplified by nickel ammonium. No immunostaining was observed by incubating the slices with the primary antibody preabsorbed with the corresponding peptide at concentrations exceeding 10- to 300-fold antibody concentration (24 h at 4° C) or without the primary antibody (negative control). For ChemR23/ERV1 and ALXR/FPR2 no signal was observed using brain slices from knock-out mice (kindly provided by M. Perretti and S. Sozzani, University of Brescia, Italy).

Double-immunostaining was performed to identify the cells expressing IL-1β, TNF-α, ALXR, ChemR23. One brain slice was used for each cell type marker. After incubation with the primary antibodies, slices were incubated in biotinylated secondary anti-goat (for IL-1β, TNF-α and ChemR23/ERV1) or anti-rabbit antibodies (ALXR/FPR2) (1:200, Vector Labs), then in streptavidin–horseradish peroxidase and the signal was revealed with tyramide conjugated to Fluorescein using TSA amplification kit (NEN Life Science Products, Boston, MA, USA). Sections were subsequently incubated with the following primary antibodies: mouse anti-GFAP (1:2500, Chemicon, Temecula, CA, USA, #MAB3402), a marker of astrocytes, or rat anti-CD11b (1:1000, MAC-1, Serotec; #MCA719, Clone 5C6), a marker of microglia, or mouse anti-NeuN (1:1000, Chemicon, #MAB377), a marker of neurons, or rabbit anti-NPY (1:25000, Peninsula Lab., CA, USA, #T-4070) to identify interneurons. Moreover, a double-staining analysis of IL-1β with ALXR/FPR2 was performed to study if they colocalized in the same cell population. Fluorescence

was detected using anti-mouse, anti-rat or anti-rabbit secondary antibody conjugated with Alexa546 (1:250; Molecular Probes, Leiden, The Netherlands, #A-11030).

Slide-mounted sections were examined with an Olympus Fluoview laser scanning confocal microscope (microscope BX61 and confocal system FV500) using excitations of 488 nm (Ar laser), 546 nm (He-Ne green laser) for fluorescein and Alexa546, respectively. The emission of fluorescent probes was collected on separate detectors. To eliminate the possibility of bleed-through between channels, the sections were scanned in a sequential mode.

Human subjects

The cases included in this study were obtained from the archives of the Departments of Neuropathology of the Academic Medical Center (AMC, Amsterdam, The Netherlands) and the VU University medical center (VUmc, Amsterdam, The Netherlands). A total of 7 hippocampal specimens (removed from patients with temporal lobe epilepsy with hippocampal sclerosis (TLE-HS) undergoing surgery for drug-resistant epilepsy) and 7 hippocampal specimens obtained at autopsy from patients who died after SE were examined. Control material was obtained at autopsy from 6 age-matched control patients, without a history of seizures or other neurological diseases. All autopsies were performed within 24 h after death. Tissue was obtained and used in accordance with the Declaration of Helsinki and the AMC Research Code provided by the Medical Ethics Committee. All cases were reviewed independently by two neuropathologists and the classification of hippocampal sclerosis was based on analysis of microscopic examination as described by the International League Against Epilepsy (Blumcke *et al.*, 2013). The clinical information of each patient is reported in Suppl. Table 1.

ALXR/FPR2 and ChemR23/ERV1 immunohistochemistry in human tissue

Human brain tissue was fixed in 10% buffered formalin and embedded in paraffin. Tissue was sectioned at 5 µm, mounted on pre-coated glass slides (Star Frost, Waldemar Knittel, Braunschweig, Germany) and processed for immunohistochemical staining (Suppl. Fig. 2E,F). Sections were deparaffinated in xylene, rinsed in ethanol (100%, 95%, 70%) and incubated for 20 min in 0.3% hydrogen peroxide diluted in methanol. Antigen retrieval was performed using a pressure cooker in 0.01 M sodium citrate buffer (pH 6.0) at 120°C for 10 min. Slides were washed with phosphate-buffered saline (PBS), pH 7.4, and incubated overnight with primary antibody anti-ALXR/FPR2 (1:500, Novus Biologicals, CO, USA, #NLS1878) and anti-ChemR23/ERV1 (1:50, Santa Cruz, CA, USA, #sc-32652) in PBS at 4° C. For single labeling, sections were washed in PBS and then stained with a polymer based peroxidase immunohistochemistry detection kit (Brightvision plus kit, ImmunoLogic, Duiven, The Netherlands) according to the manufacturer's instructions. Sections were dehydrated in alcohol and xylene and coverslipped.

As previously done in human specimens (Ravizza and Vezzani, 2006; Ravizza *et al.*, 2008), semi-quantitative analysis of staining was done by calculating the immunoreactivity score as the product of intensity of staining x frequency of stained cells. Intensity: 0=negative, 1=weak, 2=moderate, 3=strong. Frequency: 1=single to 10%, 2=11-50%, 3>50%.

Double-labelling of ALXR/FPR2 and ChemR23/ERV1 was performed with NeuN (neuronal nuclear protein; mouse clone MAB377; Chemicon, Temecula, CA, USA; 1:2000), GFAP (monoclonal mouse, Sigma, St. Louis, MO, USA; 1:4000) or HLA-DR (mouse clone CR3/43, DAKO, Glostrup, Denmark; 1:400). After overnight incubation at 4° C and washing in PBS, sections were incubated with secondary antibodies, Alexa Fluor 488 donkey anti-mouse IgG (H+L) and Alexa Fluor 568 goat anti-rabbit IgG (H+L) (1:200, Invitrogen, Eugene, OR, USA) for two hours at room temperature. Sections were coverslipped using Vectashield with DAPI (Vector Laboratories, Peterborough, UK). Fluorescent microscopy was performed using Leica Confocal Microscope TSC SP8 X (Leica, Son, The Netherlands).

Histological analysis and quantification of neuronal cell loss, neurogenesis and glia activation in mouse brain

Analyses were performed in the hippocampus ipsilateral to the injected amygdala and in the injected amygdala since in this epilepsy model the histopathology is mostly present in the injected hemisphere (Mouri *et al.*, 2008; Iori *et al.*, 2017). Mice were randomly selected in the various experimental groups for histological analysis (Suppl. Fig 4 and Suppl. 5).

Nissl or Fluoro-Jade (FJ) staining were performed to assess neuronal cell loss and degenerating neurons, respectively. Immunohistochemistry was done to analyse astrocytes (S100β) and microglia (Iba-1), and neurogenesis was assessed using doublecortin (DCX). Serial coronal sections were cut on a cryostat throughout the septo-temporal extension of the mouse hippocampus (Franklin and Paxinos, 2008) (-0.94 to -3.64 mm *from bregma*). Four series of 16 sections each were prepared and in each series the slices were stained as follows: the 1st slice Nissl, the 2nd FJ, the 3rd DCX, the 4th S100β and the 5th Iba-1. Immunohistochemical analysis of 3 brain slices per mouse (-1.34, -1.46, -1.58 mm *from bregma*) and quantification procedures were performed by two independent expert investigators blind to the identity of the samples.

Neuronal cell loss was quantified by reckoning the number of Nissl-stained neurons in the basolateral amygdala and the hilar interneurons using a digitized image of the whole hemisphere captured at 20X magnification (Virtual Slider Microscope; Olympus, Germany). Neuronal cells in CA1, CA3 pyramidal layers were too dense to allow for a sound quantification by cell counting therefore we measured the Nissl-positive area as previously described (Iori *et al.*, 2013). High-power non-overlapping fields of the whole hippocampus (20X magnification; Olympus) were

acquired to measure the total area (μm^2) occupied by Nissl-stained neurons along the CA1 and CA3 pyramidal cell layers which reflects neuronal density in each region, using ImageJ software. Data obtained in each image within the same hippocampal subfield were added together providing one single value *per* slice in each mouse. Data obtained in each of the 3 slices per brain were averaged, providing a single value for each brain, and this value was used for statistical analysis.

Degenerating neurons were identified by FJ labeling and counted as previously described (Schmued *et al.*, 1997; Ravizza and Vezzani, 2006). Briefly, sections were dried in ethanol (100%, 75% and 50%) and rehydrated in distilled water. Then, they were incubated in 0.06% potassium permanganate, washed in distilled water and transferred to 0.001% FJ staining solution. Sections were then rinsed in distilled water, mounted onto gelatin-coated slides, dried, immersed in xylene and coverslipped. High-power fields (20X magnification; Olympus) along the CA1 and CA3 pyramidal cell layers, the hilus, the basolateral amygdala, the somatosensory/perirhinal cortices were acquired. Nissl-positive cells and FJ-positive neurons were marked and an automated cell count was generated using Fiji software. Data obtained in each slice/area/brain were averaged providing a single value *per* mouse, and this value was used for statistical analysis.

S100β, Iba-1 and DCX immunostaining was carried out as previously described (Ravizza *et al.*, 2008; Filibian *et al.*, 2012; Iori *et al.*, 2013). Lack of immunostaining was observed when slices were incubated with the primary antibodies preabsorbed with an excess of the corresponding peptides, or without the primary antibodies.

S100β or Iba-1-immunostained area was measured in the whole hippocampus (20X magnification) using ImageJ software. The area was expressed as positive pixels/total assessed pixels; the percentage area with the specific staining was used for subsequent statistical analysis. The quantification of the total number of S100β-immunoreactive astrocytes was carried out using an image of the whole hippocampus captured at 20X magnification (Virtual Slider Microscope; Olympus, Germany) by an investigator who identified the cells; then an automated cell count was generated using ImageJ software.

DCX-immunostained cells were counted using an image of the hilus and the surrounding superior and inferior granule cell layer captured at 20X magnification (Virtual Slider Microscope; Olympus, Germany) as previously described (Pascente *et al.*, 2016).

Data obtained in each mouse hippocampus were averaged, thus providing a single value for each mouse, and this value was used for the statistical analysis. Although this cell counting method has some limitations as compared to designed-based stereological analysis (Schmitz and Hof, 2005) the occurrence of any bias in counting should similarly affect sham and experimental mice since these samples underwent the same procedure in parallel.

Supplementary Figures and Tables

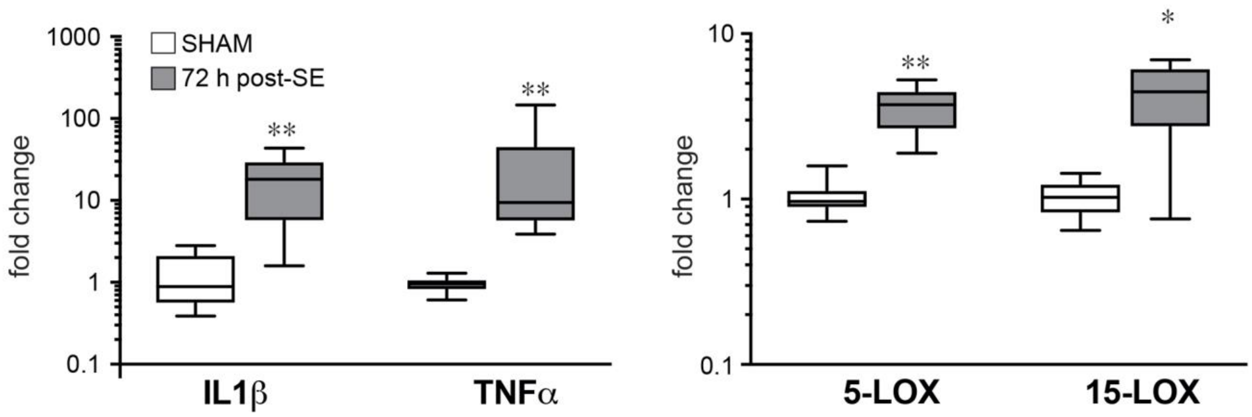

Suppl. Fig. 1. *mRNA levels of proinflammatory cytokines (IL-1β, TNF-α) and proresolving enzymes (5-LOX, 15-LOX) in the mouse hippocampus after pilocarpine-induced status epilepticus (SE)*

RT-qPCR analysis was done 72 h after SE. Reference genes are Mfsd5, Brap, Bcl2l13. Data are presented as box-and-whisker plots on a log_{10} scale depicting median, interquartile interval, minimum and maximum (n=7-10). *p<0.05, **p<0.01 vs SHAM by Mann-Whitney test.

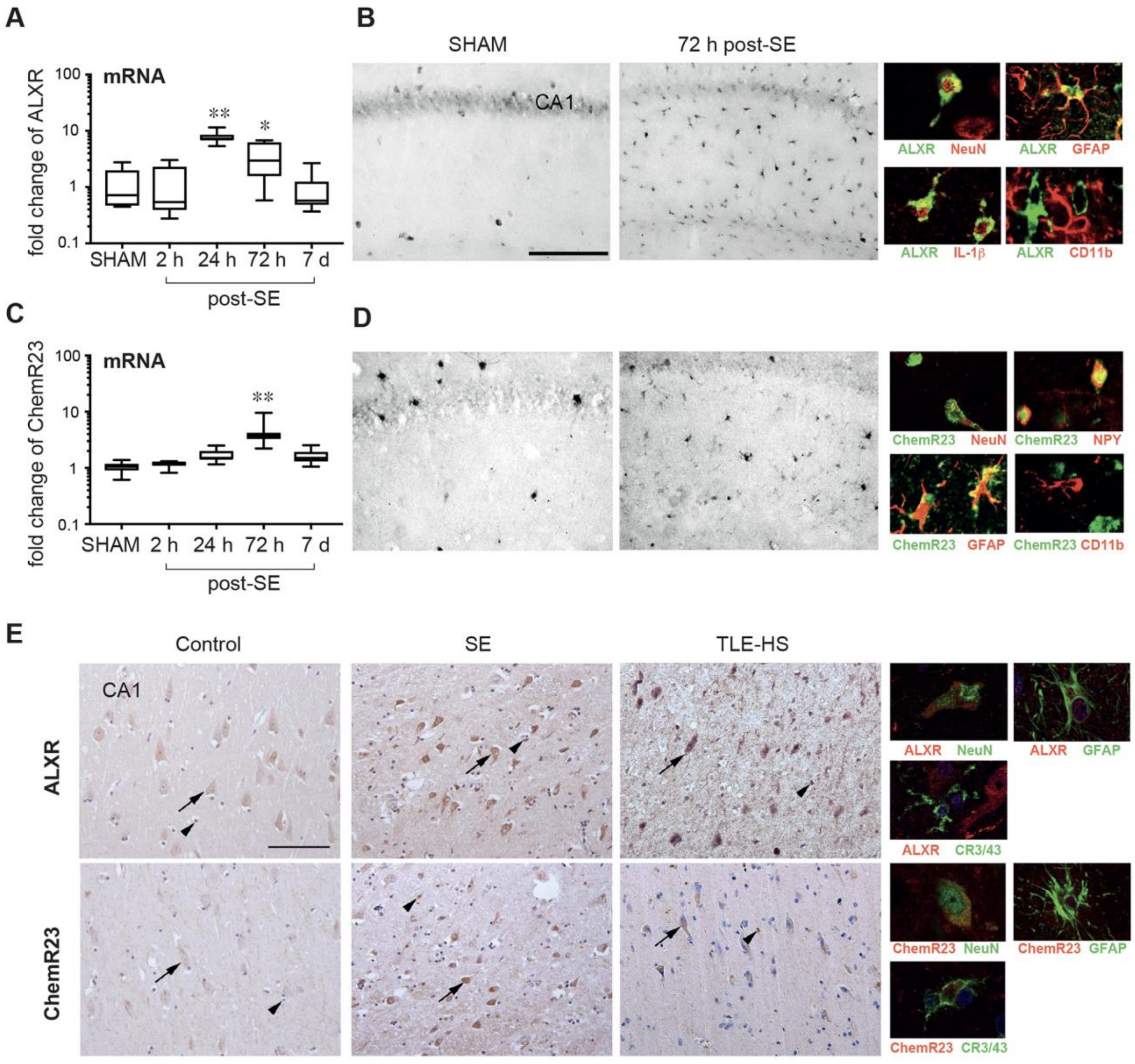

F

ALXR	CA1 neuron	CA1 glia
Control (n=6)	6	1
SE (n=7)	9 (6-9)*	6 (2-9)*
TLE-HS (n=7)	9 (6-9)*	9 (2-9)*

ChemR23	CA1 neuron	CA1 glia
Control (n=6)	6	1
SE (n=7)	9 (6-9)	6 (1-9)*
TLE-HS (n=7)	9 (6-9)*	9 (6-9)*

Suppl. Fig. 2. *mRNA and protein expression of ALXR/FPR2 and ChemR23/ERV1 in the hippocampus of mice and patients*

Mouse tissue. mRNA levels of ALXR/FPR2 (A) and ChemR23/ERV1 (C) in the hippocampus at the various time points during epileptogenesis in SE-exposed mice and in sham mice. Reference genes are Mfsd5, Brap, Bcl2l13. Data are presented as box-and-whisker plots on a $\log_{10}$ scale depicting median, interquartile interval, minimum and maximum (n=7-10). *p<0.05, **p<0.01 vs SHAM by Kruskal Wallis test with Dunn's post-hoc correction.

Panels (B) and (D) represent the immunoreactivity of ALXR/FPR2 or ChemR23/ERV1 in CA1 hippocampal area of mice 72 h post-SE and in corresponding sham mice (n=8 each group). Cells expressing ALXR/FPR2 and ChemR23/ERV1 (green) were identified by double-immunostaining

using specific neuronal (NeuN, red), astrocytic (GFAP, red), microglial (CD11b, red) and interneuron (NPY, red) markers, as indicated in the high magnification panels. ALXR/FPR2 (green) co-localized also with IL-1β (red). The double-immunostaining of ChemR23/ERV1 with IL-1β was not performed since the two primary antibodies were raised in the same host specie (goat). Co-localization signal is depicted in yellow. Scale bar 50 μm.

While ALXR/FPR2 staining was diffusely increased in the various hippocampal layers (*not shown*), ChemR23/ERV1 was predominantly induced in *strata radiatum and moleculare* of CA1.

In sham mice ALXR and ChemR23/ERV1 immunoreactivity was also detected in NeuN-positive pyramidal neurons (Suppl. Fig. 2B,D), in granule cells (*not shown*) and scattered interneurons (Suppl. Fig. 2B,D). This staining was apparently reduced after SE likely reflecting neuronal cell loss (*not shown*).

Human tissue. Panel E shows ALXR/FPR2 and ChemR23/ERV1 immunostaining in CA1 area from autoptic control tissue (n=6) and patients who died between 1 and 49 days post-SE (n=7) or patients affected by chronic epilepsy (temporal lobe epilepsy with hippocampal sclerosis; TLE-HS, n=7). We identified the cell types expressing ALXR/FPR2 and ChemR23/ERV1 (in red) by double-immunostaining using specific neuronal (NeuN, green), astrocytic (GFAP, green) and microglial (CR3/43, green) cell markers (high magnification panels). Co-localization signal is depicted in yellow. DAPI-positive nuclei are shown in blue. Scale bar 100 μm. Table (F) reports semi-quantitative analysis of staining which was done by calculating the immunoreactivity score: intensity of staining x frequency of stained cells. Intensity: 0=negative, 1=weak, 2=moderate, 3=strong. Frequency: 1≤10%, 2=11-50%, 3>50%. * $p<0.05$ vs control by Mann Whitney U test.

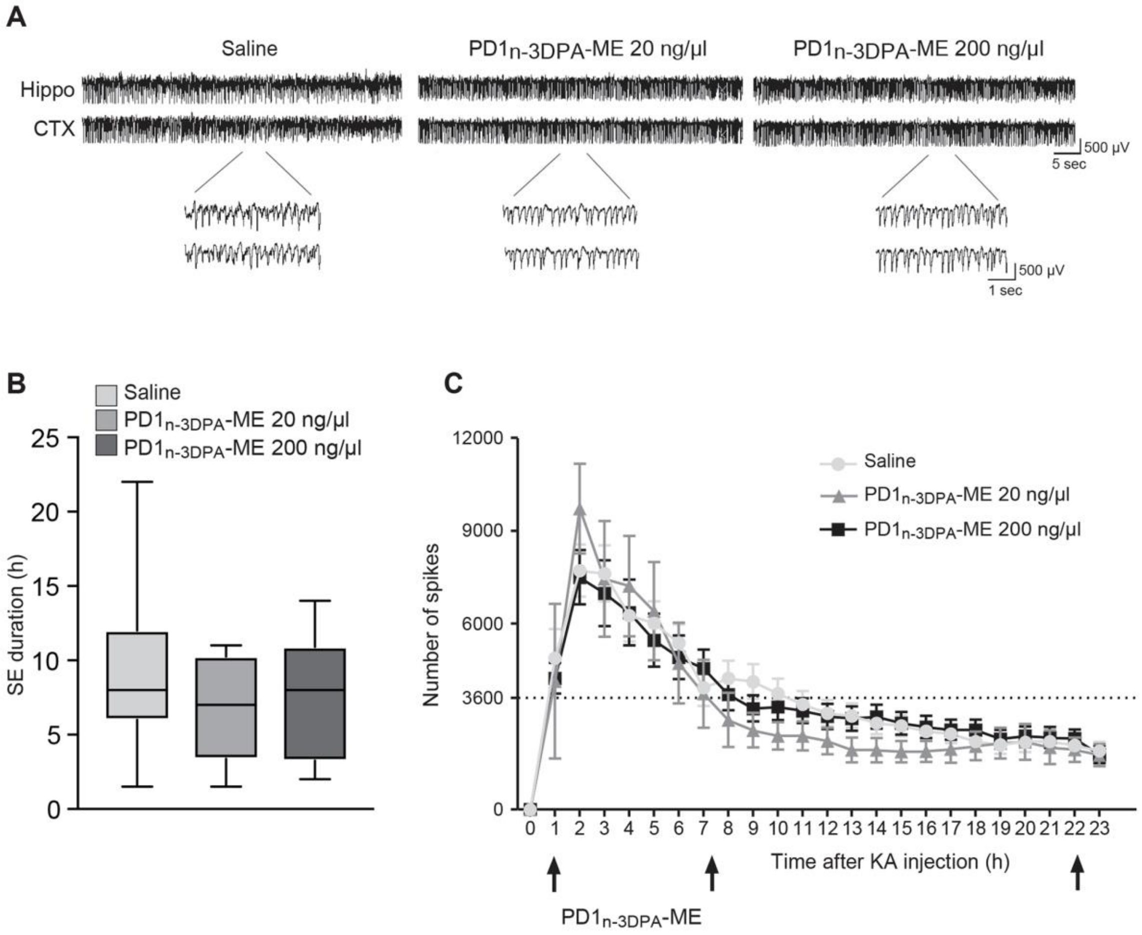

Suppl. Fig. 3. *PD1$_{n\text{-}3\ DPA}$-ME does not affect status epilepticus*

Panel A: representative EEG tracings depicting spike activity during status epilepticus (SE). Mice were EEG recorded in the hippocampus ipsilateral to the injected amygdala (Hippo) and in the contralateral cortex (CTX) of mice. Animals were treated i.c.v. with 20 ng/µl or 200 ng/µl PD1$_{n\text{-}3\ DPA}$-ME or similarly injected with saline. *Panel B* depicts the total duration of SE in the various experimental groups. The end of SE was defined by the occurrence of interspike intervals longer than 1 sec. Data are presented as box-and-whisker plots depicting median, interquartile interval, minimum and maximum: Saline (n=38); PD1$_{n\text{-}3\ DPA}$-ME 20 ng/µl (n=6); PD1$_{n\text{-}3\ DPA}$-ME 200 ng/µl (n=29). *Panel C*: Temporal spike distribution during SE in the same mice as in panel B, randomized 1 h after SE onset in vehicle or treatment groups. Each point represents the cumulative number of spikes during progressive 1 h intervals (mean ± SEM, n=number of mice). The dotted line represents the threshold number of spikes/h (3.600) below which SE ends (inter-spike intervals longer than 1 sec). Arrows indicate the time when PD1$_{n\text{-}3}$ DPA-ME or saline was injected. No difference between the experimental groups in *panel B* by Kruskal Wallis test with Dunn's post-hoc correction. Curves in *panel C* did not differ by two-way ANOVA followed by Bonferroni's multiple comparisons test.

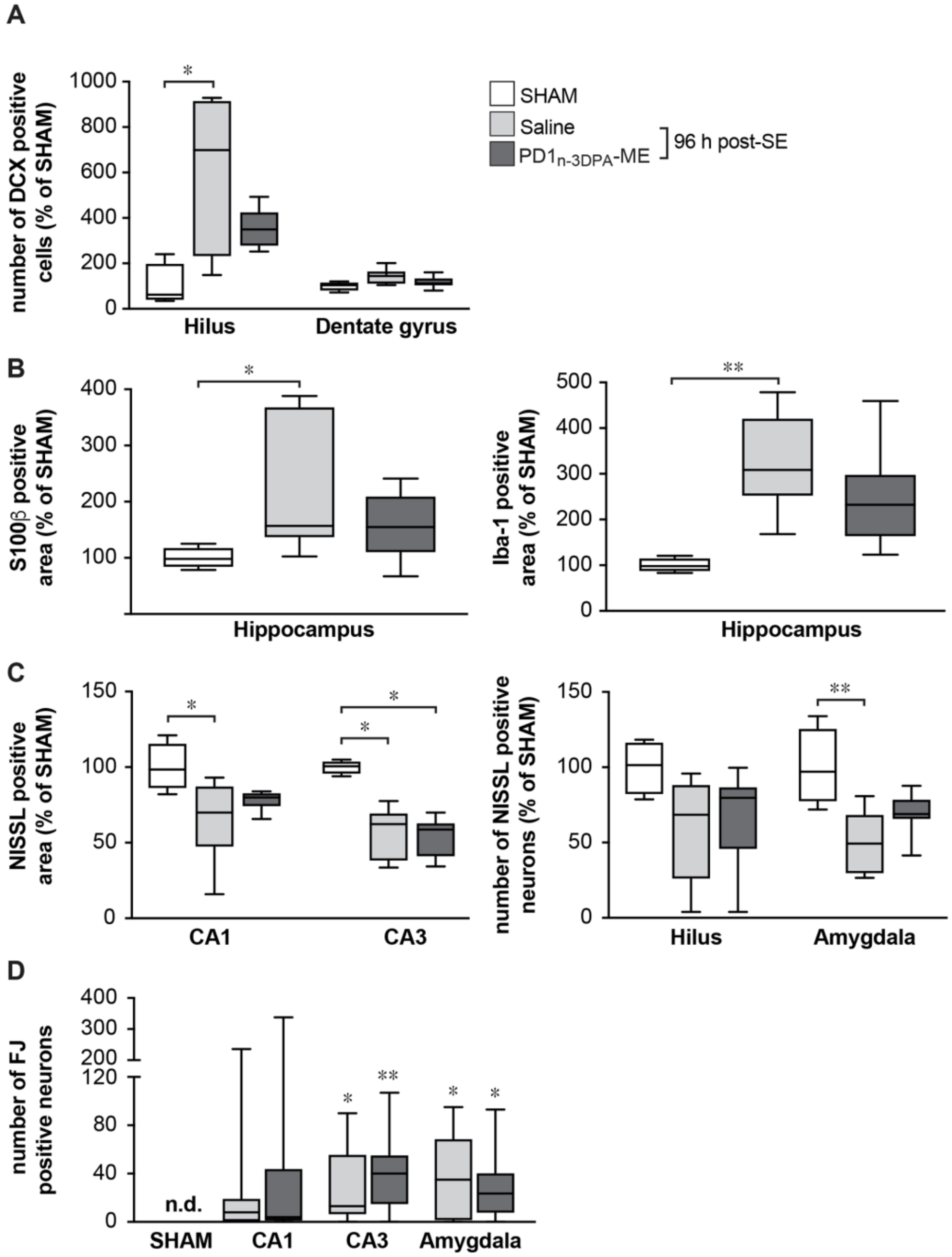

Suppl. Fig. 4. *Effect of PD1$_{n\text{-}3DPA}$-ME on neurogenesis, astrogliosis and neurodegeneration during epileptogenesis*

SE-exposed mice treated with saline or PD1$_{n\text{-}3DPA}$-ME were killed 96 h post-SE after the NORT. Mice were randomly selected in the various experimental groups. *Panel A* shows the number of doublecortin (DCX)-positive cells in the hilus and dentate gyrus; *panel B* shows S100β- and Iba-1 positive area in the hippocampus; *panel C* and *panel D* represent analysis of neurodegeneration by NISSL (C) and Fluoro Jade (FJ) staining (D). No FJ positive cells were detected in the hilus. Data are presented as box-and-whisker plots depicting median, interquartile interval, minimum and maximum (n=7-10). In panels A-C data are expressed as % change *vs* respective SHAM values. Statistical analysis was done using absolute values.. *p<0.05, **p<0.01 vs SHAM by Kruskal Wallis test with Dunn's post-hoc correction.

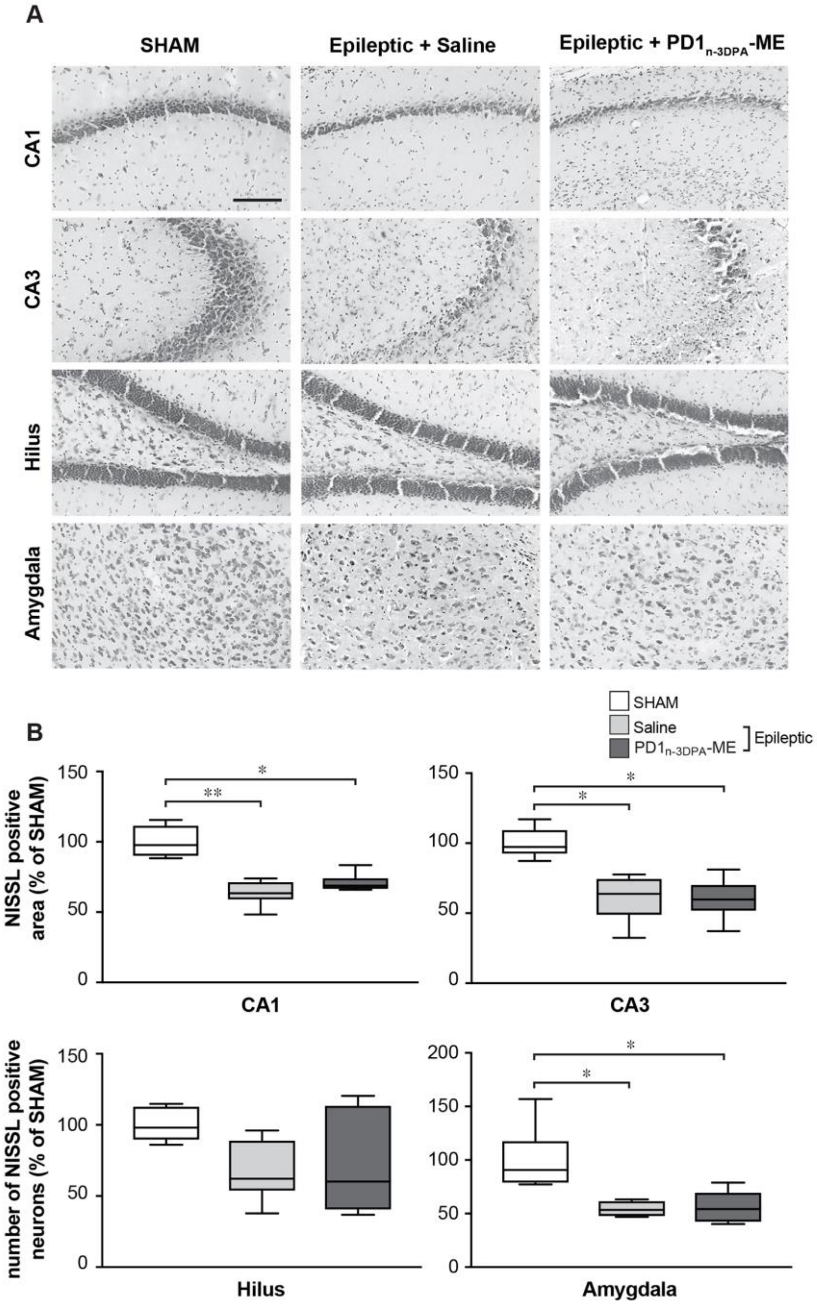

Suppl. Fig. 5. *Effect of* $PD1_{n\text{-}3DPA}$*-ME on neurodegeneration in the hippocampus and amygdala of chronic epileptic mice*

Panel A depicts representative Nissl-stained sections showing neurons in CA1 and CA3 pyramidal layers, the hilus and the amygdala of epileptic mice treated with saline or $PD1_{n\text{-}3DPA}$-ME during epileptogenesis, and in sham mice (not exposed to SE). Mice were killed at the end of EEG recordings (Figure 6). *Panel B* shows the quantification of neuronal cell loss in mice randomly selected in the various experimental groups. Data are presented as box-and-whisker plots depicting median, interquartile interval, minimum and maximum (n=6-7) expressed as % change *vs* respective SHAM values. Statistical analysis was done using absolute values. *$p<0.05$, **$p<0.01$ vs SHAM by Kruskal Wallis test with Dunn's post-hoc correction. Scale bar in *panel A*: 100 µm.

Suppl. Table 1. *Clinical characteristics of patients and controls*

case	pathology	age	gender	age of onset	seizure type	seizures/ month	AEDs	days after SE
1	control	30	m	-	-	-	-	-
2	control	75	m	-	-	-	-	-
3	control	49	m	-	-	-	-	-
4	control	35	f	-	-	-	-	-
5	control	25	f	-	-	-	-	-
6	control	31	m	-	-	-	-	-
7	SE	50	f	-	-	-	-	14
8	SE	67	m	-	-	-	-	7
9	SE	79	f	-	-	-	-	30
10	SE	58	m	-	-	-	-	3
11	SE	31	m	-	-	-	-	5
12	SE	54	f	-	-	-	-	4
13	SE	81	m	-	-	-	-	1
14	TLE-HS	29	f	13	FAS/GTCS	32	LMT,TPM	-
15	TLE-HS	57	f	47	FIAS	1	CNP	-
16	TLE-HS	37	f	20	FIAS	2	CBZ	-
17	TLE-HS	66	f	10	FIAS	8	CBZ,PB, PHT	-
18	TLE-HS	24	m	8	FIAS/GTCS	1.5	LEV,CLB,LCS	-
19	TLE-HS	49	m	8	FIAS	2	LEV,CBZ,CLB	-
20	TLE-HS	38	m	28	FIAS	6	CBZ	-

AEDs= antiepileptic drugs, CBZ= carbamazepine, CLB= clobazam, CNP= clonazepam, FAS= focal aware seizure, FIAS= focal impaired awareness seizure, GTCS= generalized tonic-clonic seizure, LCS= lacosamide, LEV= levetiracetam, LMT= lamotrigine, PB= phenobarbital, PHT= phenytoin, SE= status epilepticus, TLE-HS= temporal lobe epilepsy with hippocampal sclerosis, TPM= topiramate.

Supplementary Table 2. *Hippocampus lipid mediator profiles during epileptogenesis*

	Lipid mediator	MRM transitions		SHAM	72 h post-SE
		Q1	Q3	Mean ± SEM	Mean ± SEM
DHA-derived D-series resolvins	RvD1	375	141	0.4 ± 0.1	0.6 ± 0.1
	RvD2	375	141	1.7 ± 0.7	1.1 ± 0.2
	RvD3	375	137	4.1 ± 0.5	4.9 ± 1.2
	RvD4	375	101	8.5 ± 1.9	1.4 ± 0.3*
	RvD5	359	261	3.1 ± 1.9	4.2 ± 3.1
	RvD6	359	159	0.2 ± 0.1	0.0 ± 0.0
	17R-RvD1	375	233	1.1 ± 0.2	1.1 ± 0.2
	17R-RvD3	375	137	4.1 ± 0.5	3.3 ± 1.0
DHA-derived protectins	PD1	359	181	17.9 ± 2.3	31.2 ± 7.9
	17R-PD1	359	153	-	0.1 ± 0.1
	10S,17S-diHDHA	359	181	12.6 ± 5.8	24.6 ± 7.7
DHA-derived maresin	MaR1	359	241	14.9 ± 1.9	20.1 ± 8.8
	7S,14S-diHDHA	359	221	0.2 ± 0.2	0.3 ± 0.3

	Lipid mediator	MRM transitions		SHAM	72 h post-SE
		Q1	Q3	Mean ± SEM	Mean ± SEM
DPA-derived D-series resolvins	$RvD1_{n-3DPA}$	377	143	0.4 ± 0.1	0.5 ± 0.2
	$RvD2_{n-3DPA}$	377	233	17.4 ± 5.5	4.3 ± 1.7*
	$RvD5_{n-3DPA}$	361	263	1.1 ± 0.7	1.8 ± 0.9*
DPA-derived protectin	$PD1_{n-3DPA}$	361	183	1.2 ± 0.9	24.2 ± 8.2*
DPA-derived maresin	$MaR1_{n-3DPA}$	361	223	0.8 ± 0.5	1.1 ± 0.5

	Lipid mediator	MRM transitions		SHAM	72 h post-SE
		Q1	Q3	Mean ± SEM	Mean ± SEM
EPA-derived E-series resolvins	RvE1	349	161	1.1 ± 0.3	2.1 ± 0.5
	RvE2	333	199	1.6 ± 0.8	1.8 ± 0.6
	RvE3	333	251	4.2 ± 1.0	10.6 ± 1.8

	Lipid mediator	MRM transitions		SHAM	72 h post-SE
		Q1	Q3	Mean ± SEM	Mean ± SEM
AA-derived lipoxins	5S,15S-diHETE	335	115	74.2 ± 15.6	42.2 ± 5.4*
	$15R\text{-}LXA_4$	351	115	44.0 ± 16.9	26.3 ± 14.4
	$15R\text{-}LXB_4$	351	221	1.5 ± 0.8	4.2 ± 1.9
	LXA_4	351	115	1.5 ± 0.9	2.6 ± 2.0
	LXB_4	351	221	11.1 ± 2.5	15.7 ± 4.7
AA-derived leukotrienes	LTB_4	335	195	7.7 ± 1.7	3.0 ± 0.4*
	5S,12S-diHETE	335	195	3.9 ± 1.1	5.8 ± 3.9
	$20\text{-}OH\text{-}LTB_4$	335	195	1.4 ± 0.6	0.7 ± 0.5
AA-derived prostaglandins	PGD_2	351	189	6672.7 ± 1215.6	5655.8 ± 1538.6
	PGE_2	351	189	826.3 ± 149.6	787.9 ± 181.5
	PGF_{2a}	353	193	4331.3 ± 564.7	5563.4 ± 2154.1
AA-derived thromboxanes	TxB_2	369	169	1110.7 ± 229.3	1794.8 ± 1316.1

Lipid mediators were isolated, identified and quantified using LC-MS/MS based lipid mediator profiling. Q1, M-H (parent ion); and Q3, diagnostic ion in MS/MS (daughter ion) along with mean ± SEM values for each of the mediators (pg/exudate) identified in the 72 h hippocampus vs SHAM (n=8 mice/group). Detection limit, ~1 pg. –, below detection limit. *p<0.05 vs SHAM using Mann-Whitney test.

References

Balducci C, Beeg M, Stravalaci M, Bastone A, Sclip A, Biasini E, et al. Synthetic amyloid-beta oligomers impair long-term memory independently of cellular prion protein. Proc Natl Acad Sci USA 2010; 107: 2295–300.

Blumcke I, Thom M, Aronica E, Armstrong DD, Bartolomei F, Bernasconi A, et al. International consensus classification of hippocampal sclerosis in temporal lobe epilepsy: a Task Force report from the ILAE Commission on Diagnostic Methods. Epilepsia 2013; 54: 1315–29.

Cavalheiro EA, Silva DF, Turski WA, Calderazzo-Filho LS, Bortolotto ZA, Turski L. The susceptibility of rats to pilocarpine-induced seizures is age-dependent. Brain Res 1987; 465: 43–58.

De Simoni MG, Perego C, Ravizza T, Moneta D, Conti M, Marchesi F, et al. Inflammatory cytokines and related genes are induced in the rat hippocampus by limbic status epilepticus. Eur J Neurosci 2000; 12: 2623–33.

Filibian M, Frasca A, Maggioni D, Micotti E, Vezzani A, Ravizza T. In vivo imaging of glia activation using 1H-magnetic resonance spectroscopy to detect putative biomarkers of tissue epileptogenicity. Epilepsia 2012; 53: 1907–16.

Franklin KBJ, Paxinos G. The mouse brain in stereotaxic coordinates. Acad. Press San Diego 2008

Iori V, Iyer AM, Ravizza T, Beltrame L, Paracchini L, Marchini S, et al. Blockade of the IL-1R1/TLR4 pathway mediates disease-modification therapeutic effects in a model of acquired epilepsy. Neurobiol. Dis. 2017; 99: 12–23.

Iori V, Maroso M, Rizzi M, Iyer AM, Vertemara R, Carli M, et al. Receptor for Advanced Glycation Endproducts is upregulated in temporal lobe epilepsy and contributes to experimental seizures. Neurobiol Dis 2013; 58: 102–14.

Maroso M, Balosso S, Ravizza T, Iori V, Wright CI, French J, et al. Interleukin-1beta biosynthesis inhibition reduces acute seizures and drug resistant chronic epileptic activity in mice. Neurotherapeutics 2011; 8: 304–15.

Mazarati A, Maroso M, Iori V, Vezzani A, Carli M. High-mobility group box-1 impairs memory in mice through both toll-like receptor 4 and Receptor for Advanced Glycation End Products. Exp Neurol 2011; 232: 143–8.

Mazzuferi M, Kumar G, Rospo C, Kaminski RM. Rapid epileptogenesis in the mouse pilocarpine model: video-EEG, pharmacokinetic and histopathological characterization. Exp Neurol 2012; 238: 156–67.

Mouri G, Jimenez-Mateos E, Engel T, Dunleavy M, Hatazaki S, Paucard A, et al. Unilateral hippocampal CA3-predominant damage and short latency epileptogenesis after intra-amygdala microinjection of kainic acid in mice. Brain Res. 2008; 1213: 140–151.

Okun E, Griffioen K, Barak B, Roberts NJ, Castro K, Pita MA, et al. Toll-like receptor 3 inhibits memory retention and constrains adult hippocampal neurogenesis. Proc Natl Acad Sci USA 2010; 107: 15625–30.

Pascente R, Frigerio F, Rizzi M, Porcu L, Boido M, Davids J, et al. Cognitive deficits and brain myo-Inositol are early biomarkers of epileptogenesis in a rat model of epilepsy. Neurobiol Dis 2016; 93: 146–155.

Racine RJ. Modification of seizure activity by electrical stimulation. II. Motor seizure. Electroencephalogr Clin Neurophysiol 1972; 32: 281–94.

Ravizza T, Gagliardi B, Noe F, Boer K, Aronica E, Vezzani A. Innate and adaptive immunity during epileptogenesis and spontaneous seizures: evidence from experimental models and human temporal lobe epilepsy. Neurobiol Dis 2008; 29: 142–60.

Ravizza T, Vezzani A. Status epilepticus induces time-dependent neuronal and astrocytic expression of interleukin-1 receptor type I in the rat limbic system. Neuroscience 2006; 137: 301–8.

Schmitz C, Hof PR. Design-based stereology in neuroscience. Neuroscience 2005; 130: 813–31.

Schmued LC, Albertson C, Slikker W. Fluoro-Jade: a novel fluorochrome for the sensitive and reliable histochemical localization of neuronal degeneration. Brain Res 1997; 751: 37–46.

Turski WA, Cavalheiro EA, Schwarz M, Czuczwar SJ, Kleinrok Z, Turski L. Limbic seizures produced by pilocarpine in rats: behavioural, electroencephalographic and neuropathological study. Behav Brain Res 1983; 9: 315–35.
